# A defective interleukin-17 receptor A1 causes weight loss and intestinal metabolism-related gene downregulation in Japanese medaka, *Oryzias latipes*

**DOI:** 10.1038/s41598-021-91534-3

**Published:** 2021-06-08

**Authors:** Yo Okamura, Hiroshi Miyanishi, Masato Kinoshita, Tomoya Kono, Masahiro Sakai, Jun-ichi Hikima

**Affiliations:** 1 Interdisciplinary Graduate School of Agriculture and Engineering, University of Miyazaki, Miyazaki, Japan; 2 Department of Marine Biology and Environmental Science, Faculty of Agriculture, University of Miyazaki, Miyazaki, Japan; 3 Division of Applied Biosciences, Graduate School of Agriculture , Kyoto University, Kyoto, Japan; 4 Department of Biochemistry and Applied Biosciences, Faculty of Agriculture , University of Miyazaki, Miyazaki, Japan

**Keywords:** Immunology, Molecular biology

## Abstract

In the intestine, the host must be able to control the gut microbiota and efficiently absorb transiently supplied metabolites, at the risk of enormous infection. In mammals, the inflammatory cytokine interleukin (IL)-17A/F is one of the key mediators in the intestinal immune system. However, many functions of IL-17 in vertebrate intestines remain unclarified. In this study, we established a gene-knockout (KO) model of IL-17 receptor A1 (IL-17RA1, an IL-17A/F receptor) in Japanese medaka (*Oryzias latipes*) using genome editing technique, and the phenotypes were compared to wild type (WT) based on transcriptome analyses. Upon hatching, homozygous IL-17RA1-KO medaka mutants showed no significant morphological abnormality. However, after 4 months, significant weight decreases and reduced survival rates were observed in IL-17RA1-KO medaka. Comparison of gene-expression patterns in WT and IL-17RA1-KO medaka revealed that various metabolism- and immune-related genes were significantly down-regulated in IL-17RA1-KO medaka intestine, particularly genes related to mevalonate metabolism (*mvda*, *acat2*, *hmgcs1*, and *hmgcra*) and genes related to IL-17 signaling (such as *il17c*, *il17a/f1,* and *rorc*) were found to be decreased. Conversely, expression of genes related to cardiovascular system development, including *fli1a*, *sox7*, and *notch1b* in the anterior intestine, and that of genes related to oxidation–reduction processes including *ugp2a*, *aoc1*, and *nos1* in posterior intestine was up-regulated in IL-17RA1-KO medaka. These findings show that IL-17RA regulated immune- and various metabolism-related genes in the intestine for maintaining the health of Japanese medaka.

## Introduction

The intestinal tract is an important organ for the absorption of nutrients. Digestive enzymes, such as lipase, peptidase, and trypsin, are secreted from the pancreas or intestinal epithelial cells into the intestinal lumen to metabolize lipids and proteins present in the ingested food into absorbable metabolites. Gut microbiota are important for digestion and metabolism, and for nutrient conversion and absorption^1^. Additionally, the intestinal microbiota is important for excluding pathogens in physiological conditions. In general, intestinal immune homeostasis maintains the balance between pro-inflammatory effector Th cells and anti-inflammatory Tregs^2^.

In mammals, interleukin (IL)-17 plays important roles in the intestinal mucosal immune response. Currently, six family members of the IL-17 family (IL-17A–F) have been identified in mammals, and among these, IL-17A and F are known as key inflammatory cytokines that modulate gut microbiota through the induction of various inflammatory cytokines, chemokines and antimicrobial peptides (AMPs)^3^. It has previously been reported that IL-17A and F induce pro-inflammatory cytokines such as IL-1β, IL-6, and TNF-α; the chemokines CXCL1, CXCL8, and CCL20; and AMPs, including defensin and calprotectin^4,5^. In the mammalian intestinal tract, T helper 17 (Th17) cells (a subset of CD4^+^ T cells) produce IL-17 and have been reported to markedly accumulate, and it is known that both transforming growth factor β and IL-6 are essential for Th17 differentiation^6^. Other than Th17 cells, it was recently revealed that IL-17 is produced from various types of immune cells, including lymphocytes, γδ-T cells, natural killer T cells, innate lymphoid cells, neutrophils, mast cells, and macrophages^4^. In mice, the presence of the intestinal microbes segmented filamentous bacteria (SFB) is crucial for Th17 cell differentiation; the presentation of SFB antigen by intestinal dendritic cells triggers an increase in intestinal Th17 cells^7,8^. Mouse IL-17A, which is important for the induction of immune genes, induces the expression of carbohydrate response element binding protein (ChREBP) in hepatocytes^9^. The transcription factor ChREBP is overexpressed in lipogenic organs, such as the liver, intestine, and adipose tissues, and regulates the production of acetyl CoA from glucose by inducing the expression of liver-type pyruvate kinase (*PKLR*) and ATP citrate lyase (*ACYl*)^10^. Thus, IL-17A/F signaling is important for the host's metabolic activity in the intestinal tract, not only in regulating gut microbiota, but also in the direct induction of metabolism-related genes.

The IL-17 receptor (IL-17R) family comprises five members (IL-17RA–E). These receptors have conserved structural features, including extracellular fibronectin type III (fn III)-like domains and an intracellular region with structures similar to those of fibroblast growth factor, IL-17R, and Toll-IL-1R family domains. Of these members, IL-17RA has the broadest binding pattern with each IL-17 ligand. To date, IL-17RA has been shown to bind IL-17A, C, E, and F^11^. Generally, IL-17RA forms a heterodimer with IL-17RC before acting as a receptor for IL-17A or IL-17F, and before mediating signal transduction. In the intestine, IL-17RA is expressed by various types of cells, including epithelial cells, fibroblasts, keratinocytes, synoviocytes, endothelial cells, T cells, B cells, and macrophages^3^. In mammals, IL-17RA-knockout (KO) mice showed reduced expression levels of the defensin and regenerating islet-derived protein 3 genes (which encode AMPs) in their intestinal tracts, resulting in further effects on the composition of the microbiome^12^. Thus, the importance of IL-17-mediated innate immunity via IL-17RA in the mammalian intestinal tract is gradually being revealed.

Intestinal structures differ between teleosts and mammals. In mammals, Paneth cells are highly specialized secretory epithelial cells located in the small intestinal crypts, and have high ability to produce AMPs. Furthermore, microfold (M) cells are found in the gut-associated lymphoid tissues (GALT) of the Peyer’s patches in small intestine, and in the mucosa-associated lymphoid tissue (MALT) of other parts of the gastrointestinal (GI) tract in mammal. M cells initiate mucosal immune responses through the uptake and transcytosis of luminal antigens, such as microbes and particles. Conversely, Paneth cells and mature M cells are not reported in the intestinal epithelium of teleosts. Additionally, the GI tract of teleosts lacks gastric glands, and therefore does not have a low pH^13^. The intestines of teleosts have a membrane that is framed with chitin nanofibers inside the mucosal layer^14^. Based on these differences, the intestinal immune system of teleosts contributes to the maintenance of intestinal homeostasis via unique mechanisms compared to mammals.

The Japanese medaka (*Oryzias latipes*) is a small teleost fish, which has a short life cycle, and it is widely used as a model organism^15^. In our previous study, we created a KO medaka strain with mutations in the *il17a/f1*, which are equivalent to mammalian IL-17A/F, to clarify the role of medaka IL-17A/F signaling in the intestinal tract. Phenotypic analyses of the intestines of the IL-17A/F1-KO medaka revealed reduction in antibacterial molecules such as transferrin a (*tfa*) and lysozyme (*lyz*), and various digestive enzyme genes such as elastase (*ela*) and phospholipase A2 group IB (*pla2g1b*). Furthermore, the composition of the gut microbiome of IL-17A/F1-KO medaka was different from that of wild-type (WT) medaka. These previous data revealed the importance of IL-17A/F1 in controlling microbiota and for the induction of not only AMPs, but also expression of digestive enzyme encoding genes in the medaka intestine^16^. Data from further studies have also shown that bacterial infection induces the expression of teleost IL-17 ligands in the intestine^17,18^. Furthermore, recombinant teleost IL-17A/F show induced expression of immune genes, such as *IL1Β*, *IL6*, and *DEFB3* (β-defensin) in vitro, as has been observed in mammals^19–21^. Thus, reports on the functionality of IL-17 ligands in teleosts suggest that IL-17 signaling is very important for the immune responses, from teleosts to mammals. However, information related to the functionality of IL-17 receptors is quite limited, when compared with the available data for the ligands.

Here, we investigated the role and mechanism of *il17ra* by establishing an IL-17RA1-KO medaka strain. We characterized gene-expression levels and functions in IL-17RA1-KO medaka by RNA-sequencing (RNA-seq) analysis of the intestines of WT and IL-17RA1-KO medaka. The anterior and posterior intestines were separately analyzed, considering that distinct differences in IL-17 signaling-related immune responses were previously reported for teleosts^22^.

## Results

### Establishment of the IL-17RA1-KO medaka strain

**Figure 1:**
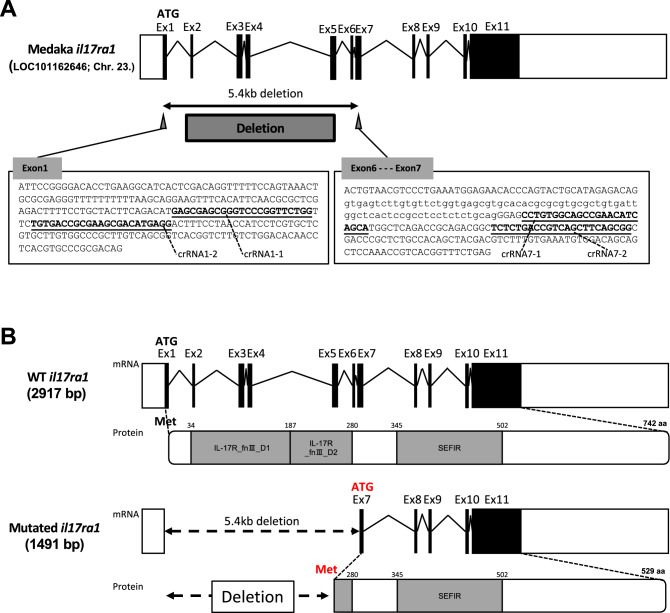
crRNA regions and comparison of the wild-type (WT) medaka interleukin-17 receptor A1 (*il17ra1*) gene with a mutated *il17ra1*. (**A**) Four crRNA regions in the medaka IL-17RA1 gene. Four crRNAs were designed against exons 1–7 of the *il17ra1* (5.4-kilobase deletion). (**B**) Comparison of mutated *il17ra1* with the WT *il17ra1*. Based on the deduced amino acid sequence, the mutated *il17ra1* lacked most of the IL-17 fn III domain, which is the extracellular domain responsible for ligand binding. The domain regions were predicted using the domain-prediction tool, SMART7. The residues of the extracellular and entire intracellular regions were the same as those of WT medaka *il17ra1*.

To KO the *il17ra1*, we deleted a genomic region spanning from exon 1 to 7 of the medaka *il17ra1* (Fig. 1A). Three of the four designed crRNAs (crRNA1-2, crRNA7-1, and crRNA7-2) showed the high mutation efficiencies (Fig. S1). A mixture of these three crRNAs was injected back into medaka embryos to edit the genome. In this manner, we established IL-17RA1-KO medaka with approximately 5.4 kb of genome sequence deleted from exon 1 to 7. The deduced amino acid sequence of mutated IL-17RA1 lacked most parts of the extracellular domain (IL-17 fn III domain) predicted using the domain search tool, SMART 7 (http://smart.embl-heidelberg.de); the IL-17 fn III domain is important for binding IL-17 ligands (Fig. 1B). After microinjection, we obtained six mutant lines (KO line A–D, J, and K; Fig. S2A) with the same open-reading frame (ORF), as the 5′ deletion occurred upstream of *il17ra1* start codon. Specifically, the *il17ra1* deletion started in the middle of the crRNA1-2 region (upstream of the original *il17ra1* start codon), and exons 1 to 6 were completely lost in each case (Fig. S2B). At the amino acid level, the deletion corresponded to most of the extracellular IL-17 fn III domain, with the downstream amino residues causing no codon frame shift (Fig. S2C). The ORF amplicon of WT *il17ra1* was not amplified from the cDNA samples of IL-17RA1-KO medaka, whereas the remaining common region (1,491 bp) was amplified, and its sequence was confirmed by sequencing (Fig. S3A). Furthermore, qPCR analysis using a primer set designed against the common region of WT and mutated *il17ra1* confirmed significantly lower expression in the posterior intestine of IL-17RA1-KO medaka (Fig. S3B).

### Weight loss in IL-17RA1-KO medaka

**Figure 2:**
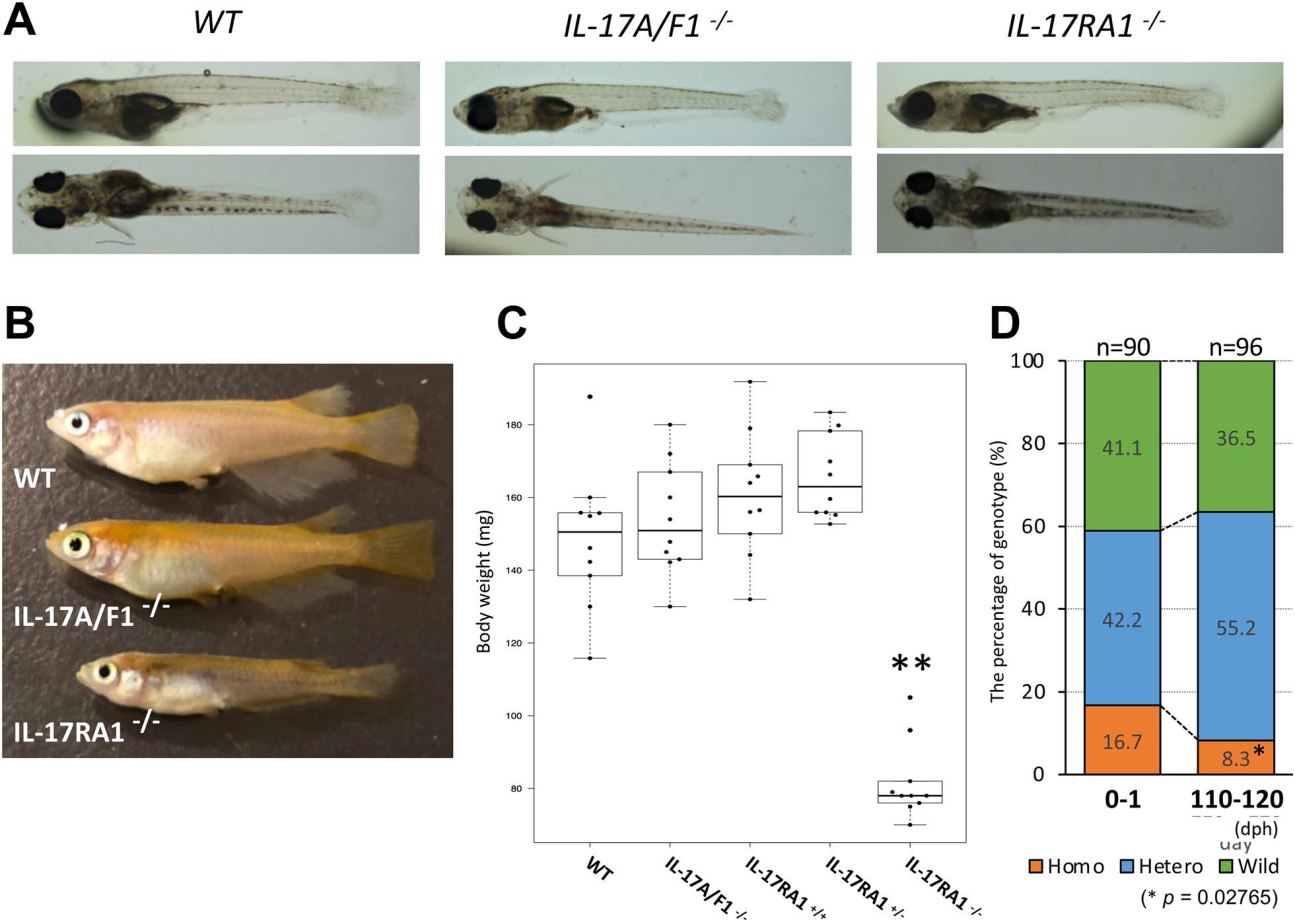
IL-17RA1-knockout (KO) medaka showed significant weight loss compared with WT and IL-17A/F1-KO medaka. (**A–C**) Comparison of medaka larvae at 0–1 day post-hatching (dph). No apparent differences were observed among WT, IL-17A/F1-KO, and IL-17RA1-KO medaka larvae at 0–1 dph (**A**), but severe weight loss was seen at 110–120 dph in IL-17RA1-KO medaka (**B**). Body weights were significantly lower only in homozygous IL-17RA1-KO medaka, compared to those of WT medaka, medaka with homozygous IL-17A/F1 mutants, and medaka with other genotypes. Different letters abovethe bars indicate significant difference at *P* < 0.05 as indicated by Tukey–Kramer multiple comparison tests in R (version 3.5.3) after one-way ANOVA. The data shown were obtained in one experiment with ten individual fish (n = 10). (**D**) The proportion of homozygous IL-17RA1-KO medaka decreased significantly at 110–120 dph. The exact binomial test was used for statistical analysis in R (version 3.5.3).

The established IL-17RA1-KO medaka line was bred with heterozygous medaka, and genotyping by the HMA method was used to confirm each generation (Fig. S2D). It was impossible to maintain the IL-17RA1-KO medaka lines by mating homozygous mutants. Homozygous individuals in all KO lines were lean, and the females did not lay eggs. Of the six IL-17RA1-KO lines, KO line C was chosen as the main line because its breeding and proliferation were more stable. From the obtained heterogeneous mate of F1 in line C, a total of 42 descendants were obtained in 60 dph (the number of each genotype was as follows, WT: 12, heterozygous: 25, homozygous: five), and the number of obtained descendant and its portion of the sum of heterozygous and homozygous were highest in the six IL-17RA1 KO lines (data not shown). Genotype comparisons were performed between WT and mutant (heterozygous and homozygous) populations immediately after hatching (at 0–1 day post-hatching [dph]) and at 110–120 dph. Furthermore, we performed a genotype comparison with our previously established IL-17A/F1-KO medaka line. No abnormalities were observed at 0–1 dph in IL-17RA1-KO medaka (Fig. 2A). However, at 110–120 dph, only the homozygous mutants of IL-17RA1-KO presented significant decreases in body weight (Fig. 2B,C), which were also observed in all obtained homozygous-mutant KO lines of IL-17RA, other than line C (i.e., lines A and K; Fig. S4). Furthermore, the proportion of surviving homozygous mutants was significantly lower at 110–120 dph than at 0–1 dph (Fig. 2D).

**Figure 3:**
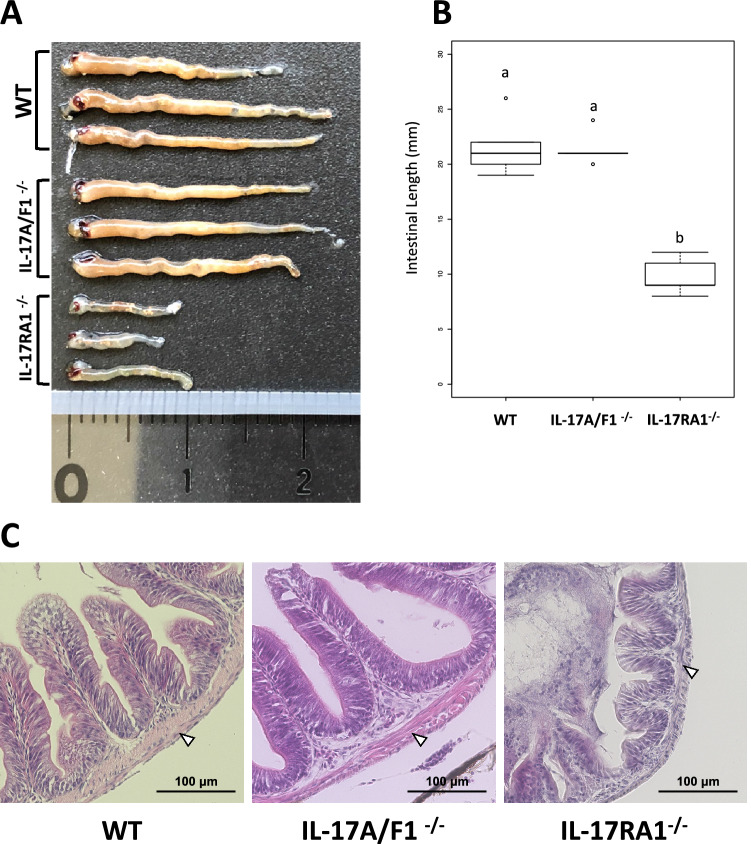
Comparisons of apparent differences in the intestinal tracts of WT, IL-17A/F1-KO, and IL-17RA1-KO medaka. (**A,B**) Comparison of the intestinal tract length. The intestinal tract of IL-17RA1-KO medaka was significantly shorter than that of WT and IL-17A/F1-KO medaka. (**C**) Hematoxylin and eosin staining of the anterior intestine. Histological changes in intestinal villus tissue were not observed in the intestinal tract of IL-17A/F1-KO medaka. However, thinner muscle layers under the intestinal villus were observed in IL-17RA1-KO medaka. The observed villus tissues of the intestinal tract were sampled from the anterior intestinal section of a 4-month-old adult fish. Different letters abovethe bars indicate significant difference at *P* < 0.05 as indicated by Tukey–Kramer multiple comparison tests in R (version 3.5.3) after one-way ANOVA. The data shown were obtained in one experiment with five individual fish (n = 5).

Notably, we observed that the intestinal tract of IL-17RA1-KO medaka was significantly shorter, than that of the WT and IL-17A/F1-KO medaka (Fig. 3A,B). Histological observation of HE-stained sections revealed dramatically thinner muscle layers under the intestinal epithelium in IL-17RA-KO medaka than that in WT and IL-17A/F1-KO medaka (Fig. 3C).

### Medaka anterior intestines expressed genes related to lipid-metabolism pathways, as observed in mammalian small intestines

**Figure 4:**
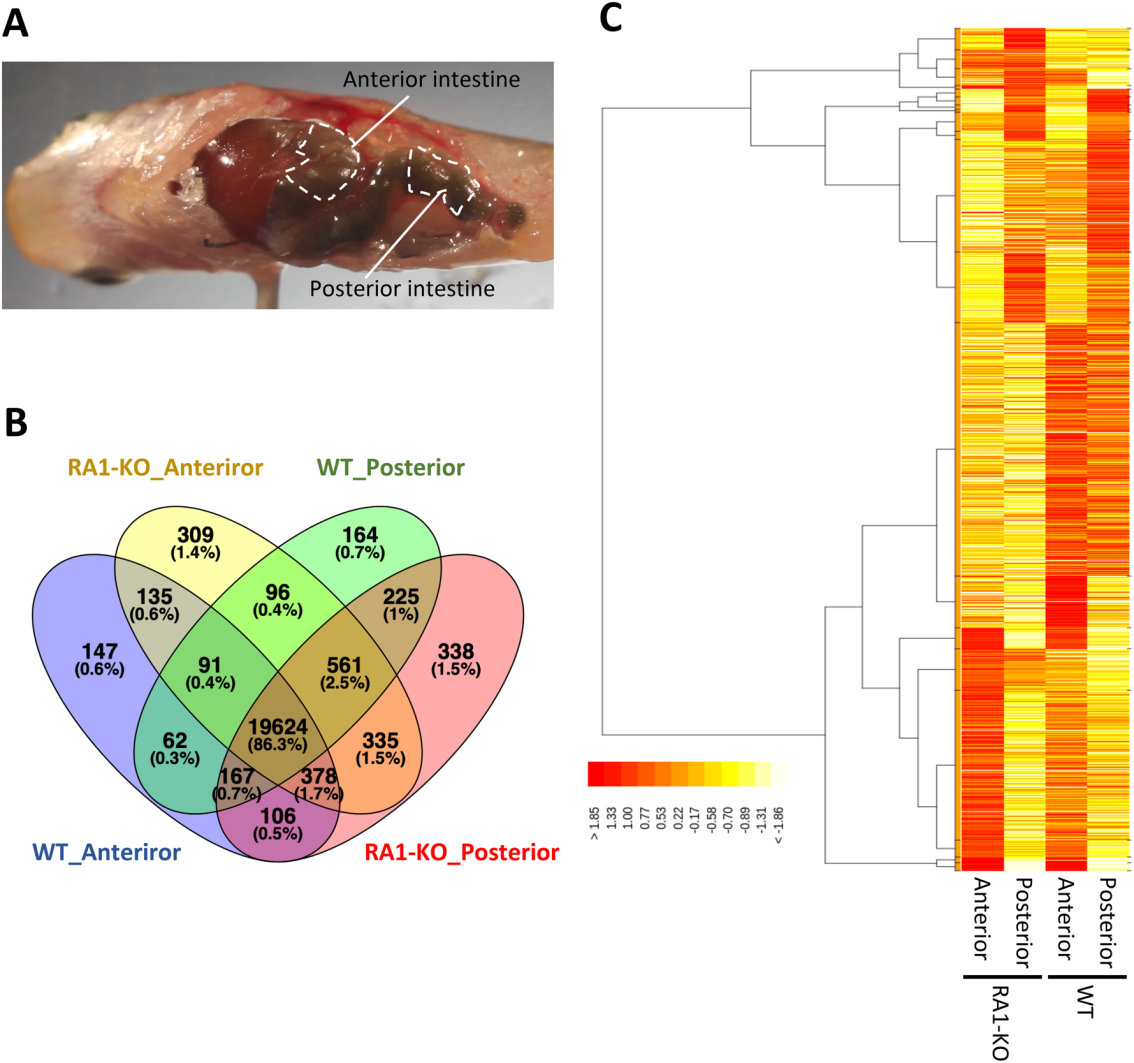
RNA-sequencing (RNA-seq) analysis of samples of the anterior and posterior intestines of WT and IL-17RA1-KO medaka. (**A**) Complementary DNA libraries were constructed with the selected intestines, as shown in the picture, and the analyzed samples were divided into two sections (anterior and posterior). (**B,C**) The overall gene-expression patterns are shown for each group (WT_Anterior, RA1-KO_Anterior, WT_Posterior, and RA1-KO Posterior). (**B**) The Venn diagram shows the numbers of expressed and overlapping genes expressed in each group. (**C**) A heat map was also used to visualize differences in the overall gene-expression patterns among the different groups. The heat map was constructed using TCC-GUI software.

**Figure 5:**
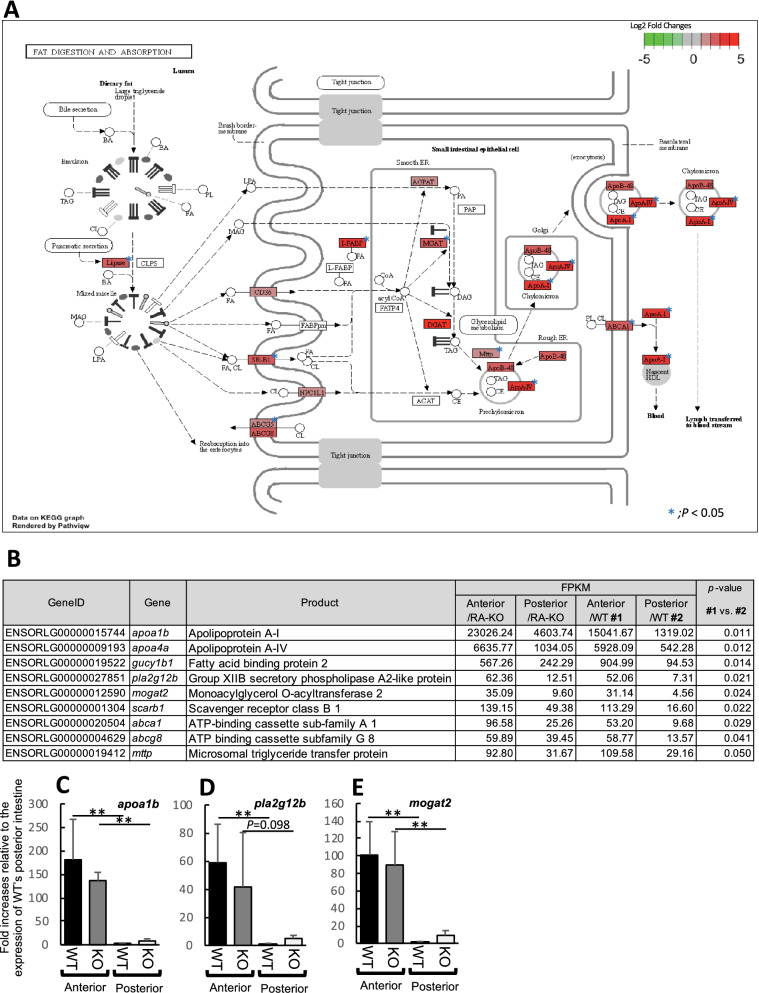
Pathway maps of notable differentially expressed gene (DEG) hits in the anterior intestines, as determined using the Kyoto Encyclopedia of Genes and Genomes (KEGG) database. (**A**) Medaka anterior intestines showed enhanced expression of genes related to the “fat digestion and absorption” (KEGG map04975) pathway, as observed in the small intestines of mammals. (**B**) Nine DEGs were significantly localized to the anterior intestine, and their expression patterns were observed in IL-17RA1-KO medaka. (**C–E**) Of the nine DEGs, the expression levels of apolipoprotein A-I (*apoa1b*), group XII B secretory phospholipase A2 (*pla2g12b*), and monoacylglycerol O-acyltransferase 2 (*mogat2*) were confirmed by quantitative polymerase chain reaction (qPCR) analysis. **P < 0.01, *P < 0.05 (Student’s *t*-test or Welch *t*-test in R version 3.5.3). The data shown were obtained in one experiment with five individual fish (n = 5).

**Figure 6:**
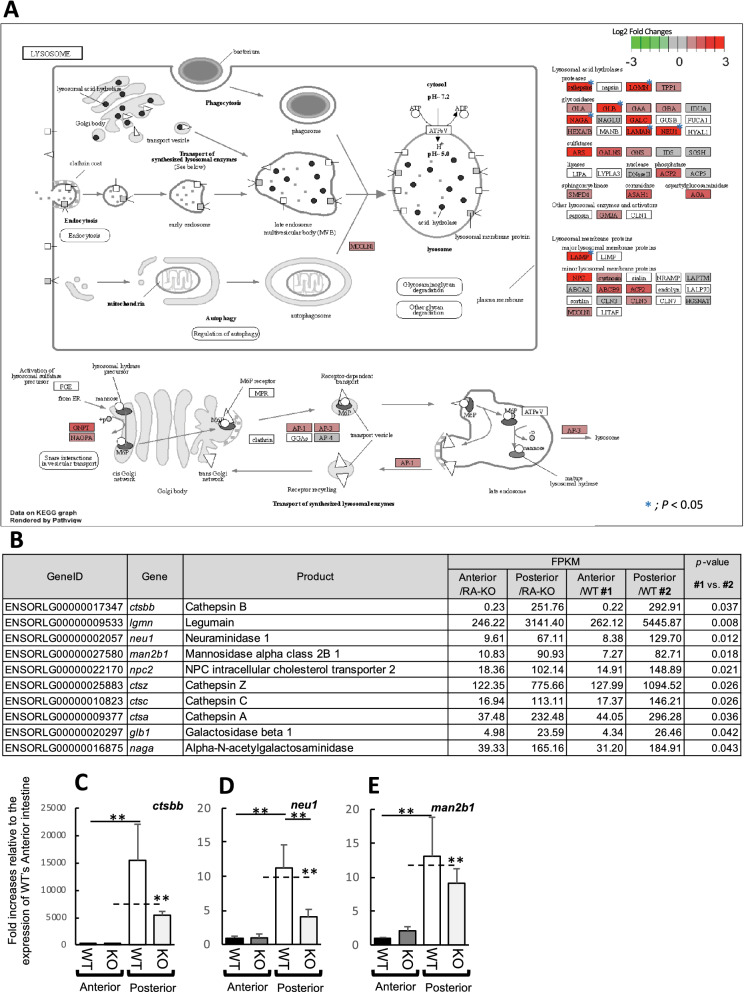
Pathway maps of notable DEGs in the posterior intestines, as determined using the KEGG database. (**A**) Medaka posterior intestines showed enhanced expression of genes related to the “lysosome” (KEGG map04142) pathway, as participated in lysosome formation. (**B**) Ten DEGs were significantly localized in the anterior intestines of WT medaka. (**C–E**) Of these 10 DEGs, the expression levels of cathepsin B (*ctsbb*), neuraminidase 1 (*neu1*), and mannosidase alpha class 2B member 1 (*man2b1*) were confirmed by qPCR analysis. **P < 0.01, *P < 0.05 (Student’s *t*-test or Welch *t*-test in R version 3.5.3). The data shown were obtained in one experiment with five individual fish (n = 5).

We next performed RNA-seq analysis to investigate functional differences between the anterior and posterior intestines of medaka, and to assess the influences of IL-17RA1 KO on downstream gene-expression levels. cDNA libraries constructed with tissue sections from anterior and posterior medaka intestines are represented in Fig. 4A. After removing low-quality reads, adaptors, and reads with a high content of unknown bases, we obtained an average of 30,840,974 (anterior intestine/WT), 30,156,147 (posterior intestine/WT), 43,545,422 (anterior intestine/IL-17RA1-KO) and 38,596,287 (posterior intestine/IL-17RA1-KO) reads from the indicated transcriptome libraries. After annotation, 20,710 genes in the WT anterior intestine, 21,529 genes in the RA1-KO anterior intestine, 20,990 genes in the WT posterior intestine, and 21,734 genes in the RA1-KO posterior intestine were detected in each library (Fig. 4B). The overall gene expression patterns were different when compared between anterior and posterior intestines, or between WT and IL-17RA1 KO when same sections of intestine were analyzed (Fig. 4C). In WT intestines, 139 genes showed higher expression levels in the anterior intestine than in the posterior intestine, and 174 genes showed significantly higher expression in the posterior intestine than in the anterior intestine. Tables S2 and S3 display the top 50 genes with large expression differences in the anterior and posterior intestines, respectively. Of the 139 genes whose expression was upregulated in the anterior intestine, 74 genes also showed significantly higher expression in the anterior intestine of IL-17RA-KO medaka than in the posterior intestine of KO medaka. Similarly, of the 174 genes that were significantly up-regulated in WT posterior intestine, 98 genes were also expressed in IL-17RA-KO posterior intestine (Fig. S5A). GO analysis of the upregulated genes suggested that the anterior intestine produced various metabolites, such as lipids, organic acids, oxoacid, and carboxylic acid. In contrast, in the posterior intestine, genes related to proteolysis and cellular catabolic processes were significantly up-regulated (Fig. S5B). Furthermore, enrichment analysis of KEGG pathways showed that the anterior intestine exhibited an enhancement of the “fat digestion and absorption” pathway of the mammalian small intestine (Fig. 5A,B). Notably, marked up-regulation of the apolipoprotein A-I (*apoa1b*), group XIIB secretory phospholipase A2-like protein (*pla2g12b*)*,* and monoacylglycerol O-acyltransferase 2 (*mogat2*) genes in both WT and IL-17RA-KO medaka was also confirmed by qPCR (Fig. 5C–E). In contrast, KEGG enrichment analysis showed that the posterior intestine exhibited enhanced expression of genes in the “lysosome” pathway (Fig. 6A,B). Of the genes with remarkably higher expression in the WT posterior intestine, up-regulation of cathepsin B (*ctsbb*), neuraminidase 1 (*neu1*), and mannosidase alpha class 2B member 1 (*man2b1*) was confirmed by qPCR (Fig. 6C–E).

### IL-17RA1-KO medaka exhibited decreased IL-17 signaling and decreased expression of various metabolism- and immune-related genes

**Figure 7:**
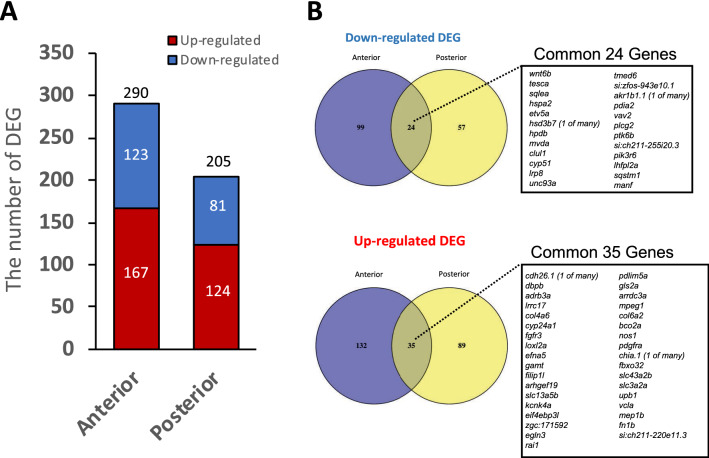
The numbers of DEGs detected in the anterior and posterior intestines of IL-17RA1-KO medaka compared to that of WT. (**A**) In the anterior intestine, 290 DEGs were identified between WT and IL-17RA1-KO. In addition, 205 DEGs were identified in the posterior intestine. (**B**) Of these DEGs, 24 genes were consistently down-regulated in both the anterior and posterior intestines of IL-17RA1-KO medaka. In addition, 35 DEGs were consistently up-regulated in both the anterior and posterior intestines of IL-17RA1-KO medaka.

**Figure 8:**
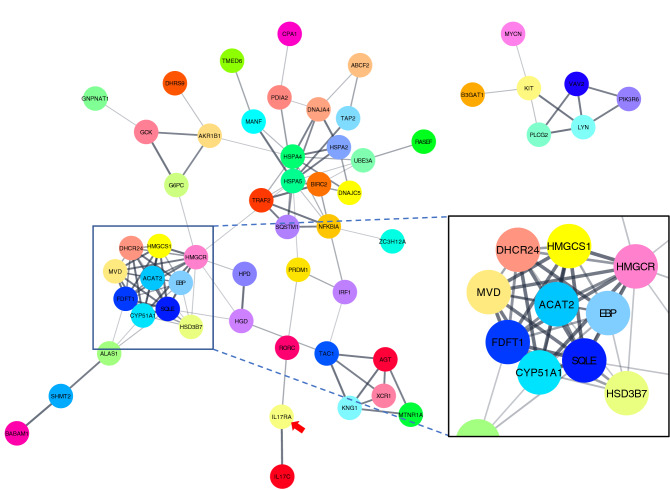
Predicted interactions between DEGs down-regulated in IL-17RA1-KO and IL-17RA medaka. The interaction network between these genes was defined using the String App database (Cytoscape). Of 123 DEGs identified, 47 formed the most complex cluster and contained IL-17RA. The gene cluster contained the ligands of IL-17RA, *il17c*, and the master transcriptional factor of IL-17-producing lymphocytes, *rorc*. Additionally, an aggregation of genes, including the mevalonate pathway-related genes, *mvda*, *acat2*, *hmgcs1*, and *hmgcr*, formed in the cluster of 47 DEGs. The red arrow points to IL-17RA.

A comparison of IL-17RA1-KO and WT medaka intestines revealed 290 DEGs with significant changes (*P* < 0.05) in the anterior intestine (up-regulated genes: 167, down-regulated genes: 123). In the posterior intestine, 205 genes were identified as DEGs (up-regulated genes: 124, down-regulated genes: 81), as shown in Fig. 7A. Of these DEGs, genes down-regulated or up-regulated by threefold are shown in Tables S4–S7. We detected 24 DEGs commonly down-regulated in both the anterior and posterior intestines of IL-17RA1-KO, as well as 35 DEGs commonly up-regulated in both intestinal regions (Fig. 7B). We further visualized interactions between DEGs using the String App in Cytoscape. We examined potential relationships between IL-17RA and the DEGs by adding IL-17RA to the analysis and localized many metabolism-related genes; 47 genes clustered together with IL-17RA among the 123 down-regulated DEGs. Interestingly, IL-17C (an IL-17RA ligand) and the Th17 master transcription factor, nuclear receptor ROR-alpha (*rorc*), were included in this relationship. Furthermore, 10 genes including mevalonate pathway-related genes, mevalonate diphosphate decarboxylase (*mvda*), acetyl-CoA acetyltransferase 2 (*acat2*), 3-hydroxy-3-methylglutaryl-CoA synthase 1 (*hmgcs1*), and 3-hydroxy-3-methylglutaryl-coenzyme A (*hmgcr*) showed particularly strong interactions (Fig. 8). These 47 genes are presented in Table 1.

**Table 1:** Differentially expressed genes (DEG) down-regulated in the anterior intestine of IL-17RA1-KO medaka and their interactions with IL-17RA, based on String App software.

Gene ID	Gene name	Gene symbol	FPKM^a^		*p* value	Fold- decrease^b^
			WT	IL-17RA1-KO		
ENSORLG00000008141	Melatonin receptor 1A	*mtnr1aa*	0.5291	0.0122	0.0203	0.023
ENSORLG00000023466	Interleukin 17C	*il17c*	1.216	0.0611	0.016	0.0503
ENSORLG00000003196	Squalene epoxidase	*sqlea*	2.796	0.4495	0.0109	0.1608
ENSORLG00000026984	Heat shock protein family A (Hsp70), member 2	*hspa2*	1.8446	0.3331	0.0333	0.1806
ENSORLG00000007442	Homogentisate 1,2-dioxygenase	*hgd*	1.1033	0.2177	0.012	0.1973
ENSORLG00000015759	Ubiquitin-protein ligase E3A	*ube3a*	1.7653	0.4081	0.0292	0.2312
ENSORLG00000016652	Chemokine (C motif) receptor 1a, duplicate 1	*xcr1a.1*	4.0532	1.0368	0.047	0.2558
ENSORLG00000002190	3 beta-hydroxysteroid dehydrogenase type 7-like	*hsd3b7 (one of many)*	27.8564	7.5955	0.005	0.2727
ENSORLG00000005554	Angiotensinogen	*agt*	3.3784	0.9213	0.0317	0.2727
ENSORLG00000012993	4-hydroxyphenylpyruvate dioxygenase-like	*hpdb*	6.4362	1.8856	0.0362	0.293
ENSORLG00000009351	Mevalonate diphosphate decarboxylase	*mvda*	8.4053	2.5234	0.0103	0.3002
ENSORLG00000009486	Nuclear receptor ROR-alpha	*rorc*	2.039	0.621	0.0446	0.3045
ENSORLG00000010025	PR domain containing 1b, with ZNF domain	*prdm1b*	13.487	4.2061	0.0085	0.3119
ENSORLG00000014448	Ribonuclease ZC3H12A	*zc3h12a*	9.9674	3.1314	0.0077	0.3142
ENSORLG00000003479	Retinol dehydrogenase 7-like	*dhrs9 (one of many)*	15.0659	4.7656	0.0139	0.3163
ENSORLG00000013226	Acetyl-CoA acetyltransferase 2	*acat2*	23.153	7.3427	0.0085	0.3171
ENSORLG00000002010	Glucokinase	*gck*	3.214	1.0907	0.0241	0.3394
ENSORLG00000005076	RAS and EF-hand domain-containing	*rasef*	5.0748	1.8392	0.0195	0.3624
ENSORLG00000020002	Cytochrome P450, family 51	*cyp51*	277.7872	102.5114	0.0076	0.369
ENSORLG00000023013	Tachykinin precursor 1	*tac1*	3.2667	1.2556	0.0355	0.3844
ENSORLG00000017362	Farnesyl-diphosphate farnesyltransferase 1	*fdft1*	13.9791	5.3888	0.0126	0.3855
ENSORLG00000012898	3-hydroxy-3-methylglutaryl-CoA synthase 1	*hmgcs1*	5.9028	2.2869	0.0203	0.3874
ENSORLG00000001448	Heat shock 70 kDa protein 4-like	*hspa4a*	7.0157	2.8314	0.0285	0.4036
ENSORLG00000013386	Transmembrane p24 trafficking protein 6	*tmed6*	56.5513	23.2489	0.0189	0.4111
ENSORLG00000022324	DNAJ homolog subfamily C, member 5	*dnajc5ab*	24.5142	10.498	0.0166	0.4282
ENSORLG00000010353	Aldose reductase	*akr1b1.1 (one of many)*	99.8516	43.7327	0.0181	0.438
ENSORLG00000008560	Protein disulfide-isomerase	*pdia2*	246.205	109.3952	0.0153	0.4443
ENSORLG00000017313	3-hydroxy-3-methylglutaryl-coenzyme A reductase	*hmgcra*	12.3052	5.4985	0.0242	0.4468
ENSORLG00000007166	TNF receptor associated factor 2	*traf2b*	15.6801	7.0424	0.0214	0.4491
ENSORLG00000011656	Kininogen-1	*kng1*	11.3471	5.1716	0.0488	0.4558
ENSORLG00000006886	Heat shock protein family A (Hsp70), member 5	*hspa5*	92.3296	43.1643	0.0205	0.4675
ENSORLG00000008898	BRISC and BRCA1 A complex member 1	*babam1*	6.2851	2.9557	0.0463	0.4703
ENSORLG00000017687	NFKB inhibitor alpha	*nfkbiab*	136.7409	65.0318	0.0245	0.4756
ENSORLG00000005556	Baculoviral IAP repeat-containing protein 2	*birc2*	13.2523	6.485	0.0322	0.4894
ENSORLG00000007453	DNAJ homolog subfamily A member 4	*dnaja4*	8.6528	4.3025	0.0382	0.4972
ENSORLG00000020572	Sequestosome 1	*sqstm1*	107.9882	54.4971	0.0307	0.5047
ENSORLG00000028270	Mesencephalic astrocyte derived neurotrophic factor	*manf*	10.6284	5.3821	0.0408	0.5064
ENSORLG00000001602	24-dehydrocholesterol reductase	*dhcr24*	16.5392	8.4677	0.0442	0.512
ENSORLG00000001131	Interferon regulatory factor 1	*irf1b*	572.8194	294.3095	0.0308	0.5138
ENSORLG00000011887	Glucosamine-phosphate N-acetyltransferase 1	*gnpnat1*	36.8613	19.0665	0.0449	0.5173
ENSORLG00000018711	Glucose-6-phosphatase catalytic subunit	*g6pca.2*	73.9836	38.5079	0.0345	0.5205
ENSORLG00000006618	Antigen peptide transporter 2-like	*abcb3*	61.6532	32.2189	0.0368	0.5226
ENSORLG00000004789	5-aminolevulinate synthase 1	*alas1*	71.7004	37.5533	0.0357	0.5238
ENSORLG00000011822	ATP binding cassette subfamily F, member 2	*abcf2a*	34.697	18.386	0.0435	0.5299
ENSORLG00000019810	Carboxypeptidase A1	*cpa1 (one of many)*	1383.346	743.9462	0.0374	0.5378
ENSORLG00000008139	EBP cholestenol delta-isomerase	*ebp*	139.2182	75.5733	0.0473	0.5428
ENSORLG00000005536	Serine hydroxymethyltransferase 2	*shmt2*	26.3162	14.2869	0.0471	0.5429

^a^Fragment per kilobase of exon length per million reads.

^b^Fold-decrease in the anterior intestine of IL-17RA1-KO, compared to that in WT medaka.

Of the DEGs with increased expression in the anterior intestine, 102 genes formed a cluster containing IL-17RA. In particular, a series of collagen genes showed strong interactions (Fig. S6). Furthermore, among the DEGs that were significantly decreased in the posterior intestine, some were possibly related to IL-17RA1, including IL-17A/F1, growth arrest and DNA damage inducible alpha (*gadd45a*), chymotrypsin-like elastase family member 2A (*ela2*), neuraminidase 1 (*neu1*), and signal transducer and activator of transcription 3 (*stat3*) (Fig. S7A). However, we did not find a gene cluster among the elevated DEGs that interacted with IL-17RA (Fig. S7B).

**Figure 9:**
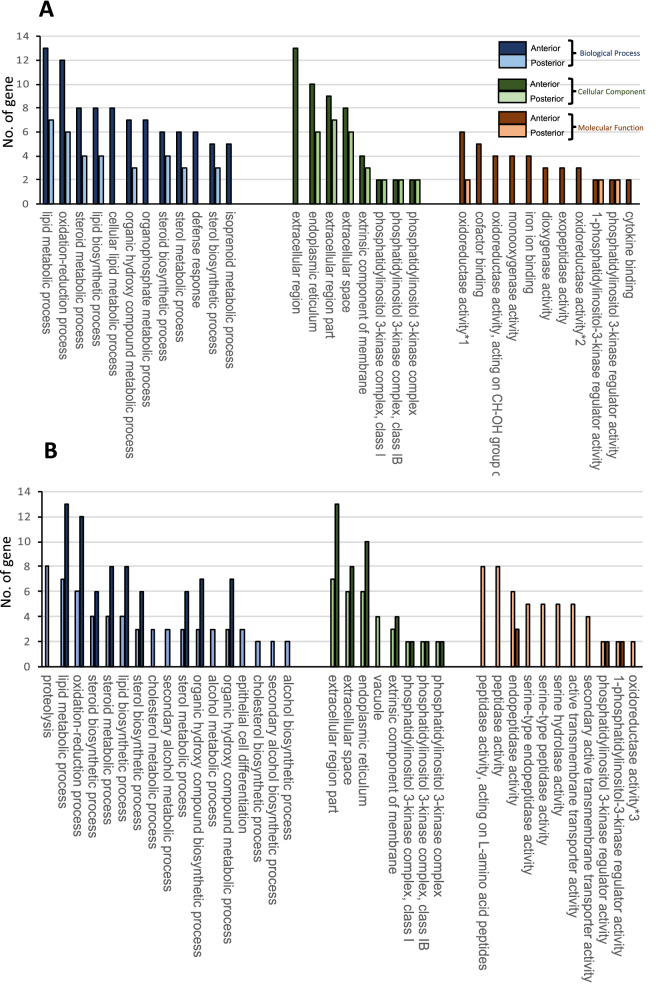
Top 12 classification of Gene Ontology (GO) enrichment of down-regulated DEGs in the anterior and posterior intestines of IL-17RA1-KO. (**A**) Down-regulated DEGs in the anterior intestine of IL-17RA1-KO medaka were related to GO terms describing various metabolic processes including lipid metabolism, steroid metabolism and lipid biosynthesis and oxidation reduction, within the category of biological processes (BPs). Immune terms such as defense response (BP category) and cytokine binding (molecular function category) were included among the top 12 terms with the most hits. (**B**) Among down-regulated DEGs in the posterior intestine of IL-17RA1-KO medaka, proteolysis was the BP term with the most hits; lipid metabolism-related and oxidation–reduction terms were also identified as hits, as observed in the anterior intestine. The numbers with asterisks indicate cases where the full-length official GO terms were abbreviated. The complete names of the official GO terms are as follows: *1: oxidoreductase activity, acting on paired donors, with incorporation or reduction of molecular oxygen, *2: oxidoreductase activity, acting on the CH-OH group of donors, NAD or NADP as acceptor, *3: oxidoreductase activity, acting on paired donors, with incorporation or reduction of molecular oxygen, NAD(P)H as one donor, and incorporation of one atom of oxygen.

Furthermore, GO analyses were performed on the DEGs using the DAVID program. Among the down-regulated DEGs in the anterior intestine of IL-17RA1-KO medaka, terms related to various metabolic process including lipid metabolism, steroid metabolism, lipid biosynthesis, and oxidation reduction were annotated in the biological process (BP) category. Of the immune terms, defense response (BP category) and cytokine binding (molecular function category) were also identified among the top 12 terms with the most hits (Fig. 9A). In contrast, among the down-regulated DEGs in the posterior intestine of IL-17RA1-KO, the most hits were obtained for proteolysis; hits were also obtained for lipid metabolism-related and oxidation–reduction terms, similar to those obtained for terms of the anterior intestine (Fig. 9B). Development of the circulatory and cardiovascular systems (Fig. S8A) were emphasized among the up-regulated DEGs in the anterior intestine of IL-17RA1-KO medaka, whereas oxidation–reduction process and organic acid metabolic process were the top two BP terms in the posterior intestine (Fig. S8B).

**Figure 10:**
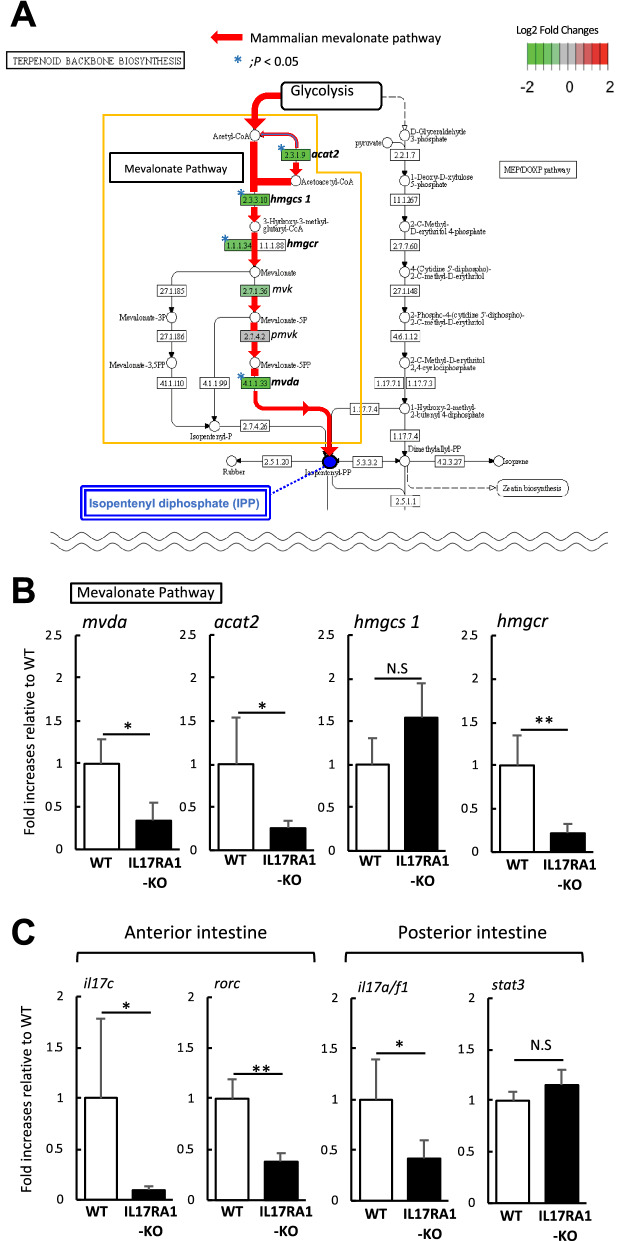
The reduction of the mevalonate pathway-related genes in IL-17RA1-KO medaka intestines. (**A**,**B**) In the anterior intestine of IL-17RA1-KO medaka; four of six enzyme-encoding genes participating in the mevalonate pathway described in KEGG map “Terpenoid backbone biosynthesis” (KEGG map00900) were significantly down-regulated as shown by RNA-seq analyses (**A**). Of these four genes, the reduction of *acat2* and *hmgcs1* was confirmed by qPCR. (**C**) qPCR analysis was also used to confirm the reduced expression of IL-17 signaling-related genes, *il17c* and *rorc*, observed by RNA-seq. **P < 0.01, *P < 0.05 (Student’s *t*-test or Welch *t*-test in R version 3.5.3). The data shown were obtained in one experiment with five individual fish (n = 5).

Among the down-regulated DEGs in the anterior intestine of IL-17RA1-KO medaka, KEGG pathway analysis revealed that four genes (*mvda*, *acat2*, *hmgcs1,* and *hmgcr*) were part of the mammalian mevalonate metabolic pathway (Fig. 10A). Down-regulated levels of *mvda, acat2* and *hmgcr* were confirmed by qPCR analysis (Fig. 10B). The levels of IL-17 signaling-related genes were also confirmed by qPCR. RNA-seq analysis showed that the anterior intestine of IL-17RA1-KO, the master transcriptional factor of IL-17-producing lymphocytes (*rorc*) and *il17c* (an IL-17RA ligand). In posterior intestine of IL-17RA1-KO, *il17a/f1* (an IL-17RA ligand) was significantly down-regulated. Conversely, *stat3* shown to be down-regulated by RNA-seq analysis in the posterior intestine of IL-17RA-KO, did not show significant changes by qPCR analysis (Fig. 10C).

### Comparison of RNA-seq results between IL-17A/F1-KO and IL-17RA1-KO medaka

Previously, we used IL-17A/F1, one of the ligands of IL-17RA knockout medaka (IL-17A/F1-KO) to reveal the role of IL-17A/F signaling in the intestine and performed RNA-seq analysis of the whole intestinal tissues (GenBank accession number: DRA008715)^16^. Comparing the down-regulated DEGs between IL-17A/F1-KO and IL-17RA1-KO medaka intestines showed that protein disulfide isomerase family A, member 2 (*pdia2*) and cytochrome P450 family 51 subfamily A, polypeptide 1 (*cyp51*) were significantly down-regulated in IL-17A/F1-KO and in both sections of IL-17RA1-KO medaka intestines. Furthermore, the anterior and posterior intestines of IL-17RA1-KO shared eight and seven down-regulated genes with IL-17A/F1-KO medaka, respectively (Table 2). Of the 17 DEGs listed in Table 2, 12 genes formed a cluster with IL-17A and IL-17RA following analysis by String App in Cytoscape (Fig. S9).

**Table 2:** Comparison of down-regulated differentially expressed genes (DEGs) in IL-17A/F1-KO and IL-17RA1-KO medaka intestines.

Gene ID	Gene symbol	Product name	Comparing to WT (log2_Fold Changes)		
			AF1-KO^a^	RA-KO (An)^b^	RA-KO (Pos)^c^
**Down-regulated genes in common between A/F1-KO, RA-KO (An), and RA-KO (Pos)**					
ENSORLG00000008560	*pdia2*	Protein disulfide isomerase family A, member 2	− 1.894	− 1.231	− 1.62
ENSORLG00000020002	*cyp51*	Cytochrome P450 family 51 subfamily A polypeptide 1	− 1.69	− 1.499	− 1.446
**Down-regulated genes in common between A/F1-KO and RA-KO (An)**					
ENSORLG00000017313	*hmgcra*	3-hydroxy-3-methylglutaryl-CoA reductase	− 4.273	− 1.223	
ENSORLG00000016356	*pglyrp6*	Peptidoglycan recognition protein 6	− 3.034	− 1.059	
ENSORLG00000019810	*cpa1*	Carboxypeptidase A1	− 2.611	− 0.956	
ENSORLG00000020058	*endou*	Poly(U)-specific endoribonuclease	− 2.597	− 1.486	
ENSORLG00000016693	*dnase1*	DNase I	− 2.159	− 1.571	
ENSORLG00000013259	*cicb*	Capicua homolog b	− 2.151	− 1.497	
ENSORLG00000007210	*tmprss13a*	Transmembrane serine protease 13a	− 2.054	− 2.116	
ENSORLG00000017611	*cyp8b2*	Cytochrome P450, family 8, subfamily B, polypeptide 2	− 2.009	− 1.501	
**Down-regulated genes in common between A/F1-KO and RA-KO (Pos)**					
ENSORLG00000010663	*ela3l*	Elastase 3 like	− 2.986		− 1.66
ENSORLG00000014439	*cel (1 of many)*	Bile salt-activated lipase	− 2.612		− 1.314
ENSORLG00000018515	*si:dkey-266f7.9*	Si:dkey-266f7.9	− 2.456		− 1.157
ENSORLG00000000965	*cpa4*	Carboxypeptidase A4	− 2.406		− 1.41
ENSORLG00000004534	*ela2*	Elastase 2	− 2.274		− 1.535
ENSORLG00000004586	*ela2l*	Elastase 2-like	− 1.972		− 1.142
ENSORLG00000014032	*nudc*	Nuclear migration protein nudC	− 1.713		− 1.658

^a^Significantly down-regulated DEGs in IL-17A/F1-KO medaka (whole intestine).

^b^Significantly down-regulated DEGs in IL-17RA1-KO medaka (anterior intestine).

^c^Significantly down-regulated DEGs in IL-17RA1-KO medaka (posterior intestine).

## Discussion

This study is the first to establish a mutant medaka line of the IL-17RA1 gene using the CRISPR–Cas9 genome-editing system, with comprehensive transcriptomic analyses. In our previous report, we revealed that medaka have two IL-17RA genes (IL-17RA1 and IL-17RA2) encoded on different chromosomes^23^. Synteny analysis suggested that the medaka IL-17RA1 is equivalent to the homologous mammalian IL-17RA gene, and qPCR analysis confirmed remarkably higher expression of IL-17RA1 than of IL-17RA2 in both the anterior and posterior intestines^23^. Thus, we selected IL-17RA1 as the target gene for further exploration in teleosts. In this study, IL-17RA1-KO medaka unexpectedly showed distinct phenotypes, unlike the IL-17A/F1-KO medaka examined in our previous study. Among the genes known to encode antibacterial molecules that were downregulated in IL-17A/F1 KO medaka, expression of Elastase encoding genes (*ela2*, *ela2l,* and *ela3*) listed in Table 2 was downregulated in IL-17RA1 KO medaka. On the other hand, expression of *tfa*, *lyz*, and *pla2g1b* was significantly downregulated in IL-17A/F1 KO medaka, but no significant decrease was observed in IL-17RA1 KO medaka. These differences might reflect the broad functionality of the IL-17RA receptor. Of the IL-17 ligand family, IL-17A, C, E, and F are known to bind IL-17RA. The binding of each ligand to IL-17RA is determined by another IL-17 receptor that can form a complex with IL-17RA (*i.e.*, heterodimers of IL-17RA with IL-17RC, IL-17RB, and IL-17RE function as receptors for IL-17A/F, IL-17E, and IL-17C, respectively). In teleosts, three genes homologous to mammalian IL-17A/F, IL-17A/F1, 2 and 3 have been identified. Furthermore, the teleost specific IL-17 ligand, IL-17N, has also been characterized, and a previous report showed that IL-17N forms cluster with IL-17A/F based on phylogenic analysis^24^. Several studies have used recombinant IL-17A/F1-3 in multiple teleost species and shown that IL-17A/F1-3 has similar bioactivities, such as enhancing NF-κB activity^25^ and inducing expression of inflammatory cytokines, chemokines and AMPs, including *il1b*, *il6*, *tnfa*, *cxcl8*, *cxcl13*, *hepcidin, defensin* and *s100a1*^17,20,21,25^. Additionally IL-17N strongly induces the expression of *il1b* and *il6*^26^. Although these recombinant proteins of IL-17A/F1-3 and N have similar ability to induce the inflammation response, the tissue distribution of each gene differed in different teleost species. In the intestines of medaka and yellow croaker, IL-17A/F1 and 3 show notably higher expression levels than IL-17A/F2^16,25^. On the other hand, the expression of trout IL-17A/F2 in the intestine is the highest among all the sampled tissues^19^. Furthermore, seabass and salmon show notably higher IL-17N expression in the brain, while expression in the intestinal expressions were comparatively low^19,27^. Most of the functional commonalities and diversities of IL-17A/F1-3 and N remain poorly understood. In mammals, IL-17C appeared to have an inflammation-inducing property (similar to IL-17A) and can induce inflammatory cytokines and AMPs such as IL-1β and defensin. However, unlike IL-17A/F, IL-17C is mainly produced by epithelial cells, rather than lymphocytes^28^. IL-17C expression has been reported in multiple teleost species, as has its ability to induce inflammatory cytokines such as IL-1β (similar to the mammalian counterpart), and up-regulated IL-17C expression has been reported during infection^27,29^. Differences in the bioactivity of IL-17A/F and IL-17C have not yet been elucidated. However, previous studies on pneumonia AECOPD, which is induced by *Haemophilus influenzae* and smoking, revealed a positive correlation between progression of the inflammatory disease and concentration of IL-17C in sputum, with no correlation with IL-17A and IL-17E^30^, suggesting that IL-17C may have unique functions compared to IL-17A.

In contrast, IL-17E (IL-25) can be produced from not only Th17 cells, but also epithelial cells, mast cells, and Th2 cells. Furthermore, IL-17E shares the lowest homology with other IL-17 families and has been implicated in the IgA, IgE, and IgG1 production, and the cytokine responses of Th2 cells^31^. However, IL-17E has not been found in teleosts. In addition, there have been limited reports on the functionality of IL-17C and IL-17E. Although IL-17RA and IL-17RD heterodimer formation has been clarified, the binding properties of these heterodimers and any associated ligand have not yet been reported. However, the intracellular site of the heterodimer can bind TRAF6, which is important for intracellular signal transduction^11,32^. Previous evidence suggests the presence of novel ligands that can signal through IL-17R. The findings of this study revealed a very interesting aspect of IL-17 ligands in immune responses, demonstrating that IL-17C and IL-17A/F1 were significantly down-regulated in IL-17RA1-KO medaka intestines. In the colon of patients with ulcerative colitis (IBD), a positive correlation exists between the mRNA levels of IL-17A and IL-17C. Furthermore, IL-17A produced from Th17 strongly induces IL-17C production from intestinal epithelial cells^33^. The marked decrease in the expression of IL-17C in IL-17RA1 KO medaka observed in this study may be a response to the disrupted IL-17A/F signaling due to IL-17RA1.

During our transcriptomic analysis, we separately examined the anterior and posterior sections of medaka intestinal tissues. The digestive system of teleosts, including medaka, is structurally different from that of mammals. Teleosts contain no organ corresponding to the mammalian stomach with low-pH gastric juice, and there is no distinct separation of the small and large intestine in the intestinal tract^13^. Our results showed that in the anterior section of the medaka intestine, the number of genes specifically expressed in the mammalian small intestine was markedly higher than that of posterior section. Previous data showed that apolipoprotein, fatty acid binding protein 2, and monoacylglycerol acyltransferase were localized in the mammalian small intestine^34–36^. In the mammalian small intestine, digestive enzymes and absorbing epithelial cells mainly perform the metabolism and absorption of dietary nutrients^37^. However, in the posterior intestine of medaka, lysosome-related genes were expressed at dramatically higher levels, when compared with their corresponding levels in the anterior section. Previous histological data showed that intestinal epithelial cells containing a large number of vacuoles are locally concentrated in the posterior intestine of multiple teleost species^38,39^.

Thus, the anterior intestine of medaka plays an important role in metabolism and nutrient absorption, similar to the small intestine of mammals. In the IL-17RA1-KO medaka, we made noteworthy observations: the reduced body weight of the homozygous mutant of IL-17RA1-KO medaka, and a significant down-regulation of various lipid metabolism-related genes in the anterior intestine. Furthermore, compared with WT medaka, shorter intestinal length and thinner intestinal tract muscle layer were observed in IL-17RA1-KO medaka. However, further investigation is required to understand the changes in intestinal structure caused by IL-17RA1 deficiency and whether these changes affect growth and health. It has been reported that serum response factor (SRF) knockout in mice causes intestinal muscle layer thinning and body weight loss^40^. Multiple reports have demonstrated that IL-17 attenuation is accompanied by body weight loss in mice. In studies using mice treated with dextran sulfate sodium to induce intestinal inflammation^41^ or infected with *Chlamydia muridarum*^42^, IL-17A attenuation using a neutralizing antibody caused more severe weight loss compared with control animals. Furthermore, the weight increase observed after feeding a high-fat diet (HFD) was slower in IL-17A-KO mice than in WT mice^43^, suggesting that IL-17A may be involved in lipid absorption. Like mammals, teleosts consume lipids as a primary energy source and excess triglycerides are stored in the liver, muscles, and adipose tissues^44^. In medaka, feeding on HFD and body weight increase showed positive correlation, as seen in mammals^45^. The lipid metabolism disorder in IL-17RA1 KO may be a major factor to cause weight loss in medaka. On the other hand, previously established KO line, IL-17A/F1 KO medaka did not show weight loss. A mutation in IL-17A/F1 alone may have less effect than in case of IL-17RA1 or mammalian IL-17A since teleosts possess three homologous gene of IL-17A/F (IL-17A/F1 to 3).

Among the metabolism-related genes with decreased expression in the anterior intestine of IL-17RA1-KO, several enzyme-encoding genes associated with mevalonate metabolism were reduced. Some human patients with mevalonate kinase (mvk) deficiency showed a significant loss of body weight^46^. In addition, the loss of adipocytes via apoptosis was reported in mice with deficient mevalonate metabolism due to the adipocyte-specific KO of 3-hydroxy-3-methylglutaryl-CoA (HMG-CoA) reductase (HMGCR)^47^. These data indicate that mevalonate metabolism is very important for the establishment of lipid tissues. Moreover, isopentenyl diphosphate (IPP) synthesized downstream of the mevalonate-metabolism pathway is very important for the activation of γδ T cells, which are known as one of main IL-17-producing lymphocytes, and IPP is directly recognized by T-cell receptors^48^. The present results indicate a causal relationship with the decreased expression of several IL-17 pathway-related genes in IL-17RA-KO medaka. Future metabolome analyses are necessary to further clarify the relationship between IL-17 signaling and lipid metabolism in greater detail. Furthermore, we are considering the prospect of performing a metagenomic analysis of IL-17RA1-KO medaka in the near future, similar to our previous study on IL-17A/F1-KO medaka. Although a previously reported study using model mice has clearly demonstrated that T cell-mediated innate immune response deficiency drastically affects intestinal microbiome composition^49^, the importance of T cell-mediated immune response with respect to gut microbiota control is largely unknown in teleosts. Moreover, it is difficult to completely understand the causes of the weight loss observed in IL-17RA1-KO medaka by only analyzing the intestinal tissue. It is also important to focus on the liver and adipose tissue (which are important for metabolism) and the brain (which controls the appetite). Additionally, DEGs based analysis was limited to searching for a common cascade effect induced by the knockout of IL-17A/F1 or IL-17RA1. PPI network analysis using WGCNA allows for the construction of modules based on the correlation of co-expression, which cannot be clarified by DEG analysis, for the visualization and determination of the hub gene in the correlation produced by this method^50,51^: WGCNA, which requires the preparation of read data from multiple individuals in each group, should be the focus of future research in this area.

In conclusion, we established an IL-17RA1-KO medaka line and presented the first report of an IL-17R-KO medaka line. IL-17RA1-KO medaka showed significant weight loss and decreased survival at 4 months after hatching. Furthermore, RNA-seq analyses revealed the downregulation of various metabolism-related genes, including mevalonate pathway- and IL-17 signaling-related genes, in the anterior intestine of IL-17RA1-KO medaka. Our results suggest that IL-17RA1 not only participates in immune responses, but also maintains intestinal homeostasis, such as lipid metabolism. However, in this study, we did not elucidate the genes encoding IL-17 ligand that bind to IL-17RA, which specifically modulate phenotypes, such as the observed weight decrease. Future studies are needed to establish KO strains of medaka for IL-17RA ligands other than IL-17A/F1 and to analyze in detail the effects of IL-17RA-mediated signal transduction on fish growth and health.

## Materials and methods

### Medaka

The Cab strain (Kyoto-Cab strain) of Japanese medaka was maintained in several transparent plastic tanks with a circulating water system (26 °C) under a 14-h light and 10-h dark cycle. For gene-expression analysis, we used 4-month-old WT and mutants (KO) fish, and weighing 100–200 mg. We performed routine water quality checks once a week: dissolved oxygen (over 5.0 mg/L), ammonia/ammonium (less than 0.1 mg/L), nitrite ion (less than 1.6 mg/L), and pH (between 6.5 and 8.0). Each medaka is fed with 10 Artemia twice a day by hand. The circulation system was turned off for 30 min during feeding, and after that, the residue was collected.

All animal experiments were conducted according to the relevant national (Act on Welfare and Management of Animals, Ministry of the Environment, Japan) and international guidelines. All animal experiments were approved by the institutional animal care and use committee, Department of Marine Biology and Environmental Sciences, University of Miyazaki, Japan (R3-002). Our research was also performed in accordance with the ARRIVE guidelines.

### Body weight measurements and histological staining

Villus tissues of the intestinal tract were sampled from the anterior section of the intestine in 4-month-old adult fish. Intestinal tissues were fixed in 4% paraformaldehyde/0.1 M phosphate buffer solution and embedded in paraffin. Sections (8 μm) were stained with hematoxylin and eosin (HE) for microscopic observations (20× magnification). WT and KO adult medaka (4-months-old, n = 10) were anesthetized using MS 222 (Sigma-Aldrich, St. Louis, MO), and the water on their bodies was wiped off before weighing the medaka on a Sartorius Analytical Balance BP121S (Sartorius, Germany).

### Establishment of IL-17RA1-KO medaka strains

Approximately 0.5 mL of a solution containing Synthetic CRISPR (cr) RNAs (25 ng/μL), trans-activating crRNA (40 ng/μL), and clustered regularly interspaced short palindromic repeats (CRISPR)-associated protein 9 (Cas9) mRNA (100 ng/μL) was injected into one- to two-cell stage embryos with a manipulator (NARISHIGE, Tokyo, Japan). Two different crRNAs were designed to target exons 1 and 7 of the medaka IL-17RA1 gene and to delete approximately 5.4 kilobases (kb) (Fig. 1A). The sequences of four crRNAs are shown in Table S1. Genomic DNA (gDNA) was extracted from randomly selected embryos to confirm gene-editing efficiencies, using the heteroduplex mobility assay (HMA). Filial generation (F) 0 fish, grown from the injected fertilized eggs, were interbred with WT medaka (Cab strain) to produce F1 heterozygotes. Among the F1 fish, males and females carrying an identical mutation were mated (in-crossed) to obtain homozygous progeny and/or a mutant line (F2). HMA was performed to detect the mutated locus in the genome, and the mutant efficiency was confirmed. The sequences of polymerase chain reaction (PCR) primers used to generate products for the HMAs are shown in Table S1. Before collecting the gDNA samples, the medaka were anesthetized using MS-222 (Sigma-Aldrich). Briefly, gDNA was prepared from embryos or scraped body surfaces by dissolving the samples in 20 μL of a solution containing 0.2 mM EDTA and 25 mM NaOH, followed by incubation at 95 °C for 20 min. The samples were neutralized with the same volume of 40 mM Tris/HCl (pH 8.0). Each gDNA-containing solution was used as a template for PCR, which was performed in 10-µL reactions containing 5 µL KOD One PCR Master Mix (TOYOBO, Osaka, Japan), 1 μL of gDNA-containing solution, 0.25 μL each of two forward primers (IL-17RAF1 and IL-17RAF2), and 0.5 μL of a reverse primer (IL-17RAR1). The primers were used at final concentrations of 5 pM. The PCR program was initially run at 94 °C for 1 min, followed by 38 cycles of 94 °C for 10 s, 66 °C for 5 s, and 68 °C for 5 s. The amplicons were separated by 12% polyacrylamide gel electrophoresis to compare their migration patterns.

### RNA extraction and complementary DNA (cDNA) synthesis for next-generation sequencing and real-time quantitative PCR (qPCR) analysis

For qPCR analysis, total RNA was extracted from the whole intestines of adult medaka using an RNAiso Plus Kit (Takara Bio Inc., Kusatsu, Japan), according to manufacturer’s instructions. For RNA-seq analysis, each intestinal section (anterior and posterior intestines) was dissected from three medaka and pooled together in the same tube (normalized). For each group, two sets of sample tubes containing three medaka intestine specimens each were subjected to RNA extraction, and equal volumes of total RNA (300 ng) from the two samples from the same group were mixed. The RNA concentrations were quantified with a NanoDrop spectrophotometer (Thermo Fisher Scientific, Waltham, MA, USA), and an optical-density ratio (260:280 nm) of 1.8 was set as the minimum quality cut-off value for RNA purity. The samples were sequenced using a Hi-Seq instrument by DNAFORM (Japan).

For qPCR analysis, RNA samples were extracted from individual medaka (n = 5), and the concentration and quality of each sample were determined as described above. cDNA was synthesized using 200 ng of total RNA extracted from each sample using the ReverTra Ace qPCR RT Master Mix with gDNA Remover (TOYOBO, Japan), according to manufacturer’s instructions.

### Sequence read mapping, differential-expression analysis, and gene-enrichment analysis

Processed reads were deposited in the DDBJ Sequence Read Archive under accession number DRA010584. Subsequently, the collected reads were mapped to the annotated medaka Hd-rR reference genome (release 85; http://www.ensembl.org/index.html) using the STAR program and further analyzed using the Feature Counts function. Transcriptional-expression values were estimated as fragments per kilobase of exon length per million reads, and transcripts with a *P* value < 0.05 were considered to be differentially expressed genes. For the calculation of *P* value, DESeq2, which is a comparative analysis tool were used and the DESeq2 were set according to the previously published protocol^52^. After identifying genes exhibiting significant expression changes for each comparison, gene-enrichment analysis was performed using the Database for Annotation, Visualization and Integrated Discovery (DAVID) program^53^. Gene Ontology (GO) terms in the biological processes (GOTERM_BP_FAT), cellular component (GOTERM_CC_FAT), and molecular function (GOTERM_MF_FAT) categories, as well as Kyoto Encyclopedia of Genes and Genomes (KEGG) pathways^54^, were selected.

### Data visualization

Differences in the gene-expression levels (log_2_ fold-changes) related to each pathway map in the KEGG database was performed using the pathview package of R: A Language and Environment for Statistical (version 3.5.3)^55^ in RStudio (version 1.2)^56^. A heat map of all gene-expression levels was constructed using the software program, TCC-GUI. Gene interactions and networks were analyzed using String App database of Cytoscape (version 3.8.0)^57^. The String App database was used to predict interactions with each differently expressed gene (DEG), based on the presence of gene fusions, neighboring genes, co-occurrence, experimental findings, text mining, database analysis, and evidence of co-expression.

### Gene-expression analysis by PCR and quantitative real-time PCR (qPCR)

qPCR was performed to analyze differences in expression patterns between WT and KO intestinal tissues. Total RNA was extracted from the intestines of medaka (n = 5 from each group), and their respective cDNAs were prepared separately as described in the above section. Target genes for expression analysis were selected based on the RNA-seq results, and the sequences of the primers used are shown in Table S1. PCR amplification was performed to quantify the expression levels of genes located in the anterior or posterior intestines in 15-μL reactions containing 7.5 μL KOD One PCR Master Mix, 1.5 μL cDNA (equally normalized cDNA from three medaka), 1.5 μL (5 pmol) each of the forward and reverse primers, and 3 μL distilled water. The PCR program was initially run at 94 °C for 1 min, followed by 25 cycles of 94 °C for 10 s, 63 °C for 5 s, and 68 °C for 5 s. qPCR amplification was conducted in triplicate in a total volume of 15 μL containing 7.5 μL Brilliant III Ultra-Fast SYBR Green QPCR Master Mix (Agilent Technologies Japan, Ltd., Hachioji, Japan), 1.5 μL cDNA (not normalized), and 1.5 μL (5 pmol) each of the forward and reverse primers. The qPCR program was run at 95 °C for 15 s and 60 °C for 30 s, followed by 40 cycles on a CFX connect (Bio-Rad Laboratories, Inc., Hercules, CA). Melting-curve analysis of the amplified products was performed after thermocycling was complete to confirm the specificity of amplification. For selection of the gene as an internal control, we investigated the aptitude of β-actin (*actb*), elongation factor 1 (*ef1α*) and glyceraldehyde 3-phosphate dehydrogenase (*gapdh*) genes using the software NormFinder^58^. As *actb* showed most stable value (Stability score 0.211 in NormFinder), we chose *actb* for internal control gene in this study. Furthermore, PCR efficiency (%) of each primer sets using qPCR analysis were calculated according to procedure described in the report^59^, and calculated efficiencies were shown in Table S1.

Relative expression ratios were calculated based on the comparative-cycle threshold (Ct) method (2^-ΔΔCT^ method). Using this method, the Ct values of target genes and the internal control were determined for each sample, after which the average Ct value from triplicate experiments was used to calculate expression levels, relative to that of *actb*. In statistical analysis, F-test was performed for checking the homogeneity of variance. For F-test, command of R (version 3.5.3), var test (for two groups comparison) and Hartley test (for four groups comparison) were used respectively. After that, student *t*-test was used when homoscedasticity could be assumed between the two groups, and Welch *t*-test was used when it could not be assumed.

## Supplementary Information

Supplementary Information 1.

Supplementary Information 2.

## Abbreviations

AMP: Antimicrobial peptide
Cas9: CRISPR-associated protein 9
CRISPR: Clustered regularly interspaced short palindromic repeats
DAVID: Database for Annotation, Visualization and Integrated Discovery
DEG: Differentially expressed gene
dph: Day post-hatching
fn III: Fibronectin type III
gDNA: Genomic DNA
GO: Gene Ontology
HE: Hematoxylin and eosin
HFD: High-fat diet
HMA: Heteroduplex mobility assay
IL: Interleukin
IL-17RA1: Interleukin-17 receptor A1
kb: Kilobase
KO: Knockout
ORF: Open-reading frame
PCR: Polymerase chain reaction
qPCR: Quantitative PCR
RNA-seq: RNA sequencing
SFB: Segmented filamentous bacteria
Th: T helper
WT: Wild-type
