# Supplementary material for: A defective interleukin-17 receptor A1 causes weight loss and intestinal metabolism-related gene downregulation in Japanese medaka, Oryzias latipes
Source: Sci Rep. 2021 Jun 8;11:12099. doi: 10.1038/s41598-021-91534-3 (PMC8187396; doi:10.1038/s41598-021-91534-3)
Supplement: Supplementary file 1 — Supplementary Information 1. [file 41598_2021_91534_MOESM1_ESM.pdf]

Supplementary information 1

A defective interleukin-17 receptor A1 causes weight loss and intestinal metabolism-related gene downregulation in Japanese medaka, *Oryzias latipes*

Yo Okamura[1], Hiroshi Miyanishi[2], Masato Kinoshita[3], Tomoya Kono[4], Masahiro Sakai[4], Jun-ichi Hikima[4]*

[1] Interdisciplinary Graduate School of Agriculture and Engineering, University of Miyazaki, Miyazaki, Japan

[2] Department of Marine Biology and Environmental Science, Faculty of Agriculture, University of Miyazaki, Miyazaki, Japan

[3] Division of Applied Biosciences, Graduate School of Agriculture, Kyoto University, Kyoto, Japan

[4] Department of Biochemistry and Applied Biosciences, Faculty of Agriculture, University of Miyazaki, Miyazaki, Japan

*Corresponding author email: jhikima@cc.miyazaki-u.ac.jp

Contents**

Table S1. crRNA and primer sequences used in this study.

Table S2. List of top 50 genes with significantly higher expression in WT anterior intestine compared to posterior intestine.

Table S3. List of top 50 genes with significantly higher expression in WT posterior intestine compared to anterior intestine.

Table S4. List of the genes down-regulated by a third-fold in the anterior intestine of IL-17RA1-KO medaka.

Table S5. List of the genes down-regulated by a third-fold in the posterior intestine of IL-17RA1-KO medaka.

Table S6. List of the genes up-regulated over 3-fold in the anterior intestine of IL-17RA1-KO medaka.

Table S7. List of the genes up-regulated over 3-fold in the posterior intestine of IL-17RA1-KO medaka.

Fig. S1. Efficiency of mutation by each crRNA.

Fig. S2. The mutation start and end point of mutated *il17ra1*.

Fig. S3. The ORF comparison of WT and mutated *il17ra1* and its gene expression level in each section of the intestine.

Fig. S4. The weight losses seen in other lines (KO lines A and K) homozygous mutant of IL-17RA1-KO medaka.

Fig. S5. The enrichment of gene ontology (GO) classification of the differently expressed genes (DEGs) comparing between anterior and posterior intestines.

Fig. S6. The prediction of the interaction among DEGs up-regulated in IL-17RA1-KO with IL-17RA.

Fig. S7. The prediction of the interaction between posterior DEGs in IL-17RA1-KO and IL-17RA.

Fig. S8. Top 12 classification of GO enrichment of up-regulated DEGs in the anterior (A) and posterior intestines (B) of IL-17RA1-KO.

Fig. S9. The prediction of the interaction among DEGs down-regulated commonly in IL-17A/F1-KO and IL-17RA1-KO with IL-17A and IL-17RA.

****Table S1-S7** were provided in the separated excel file (See **Supplementary information 2**).

Fig. S1.

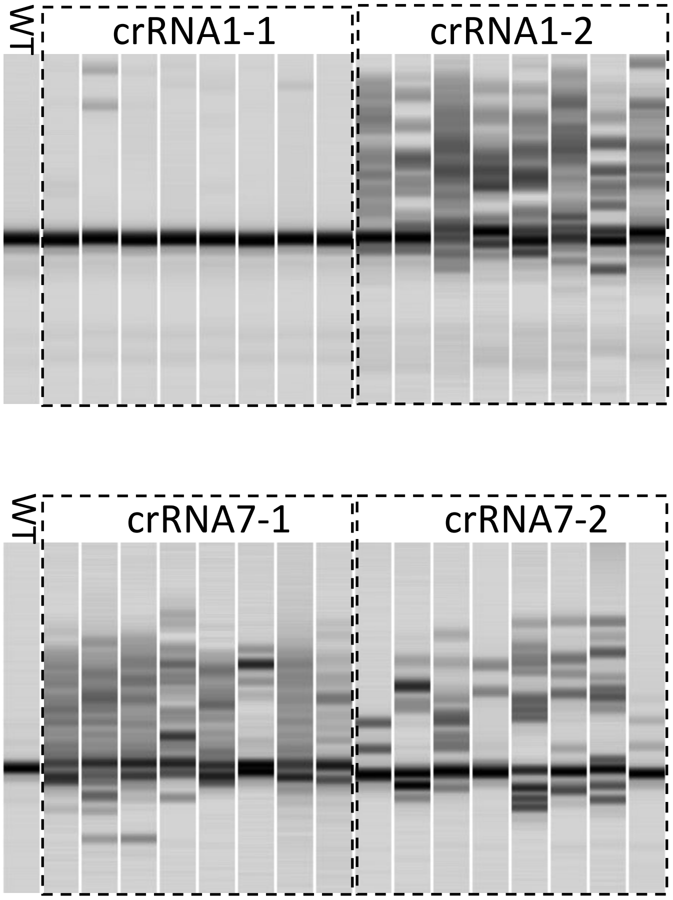

Fig. S1. Efficiency of mutation by each crRNA. The mutation efficiency was evaluated by HMA method. The separated gel lanes were visualized using the MultiNA (SHIMADZU, Japan). The results of HMA examined for the fertilized medaka eggs treated by crRNA1-1, crRNA1-2, crRNA7-1 and crRNA7-2 are shown. Of these, crRNA1-2, crRNA7-1 and crRNA7-2 showed high mutant efficiency.

Fig. S2.

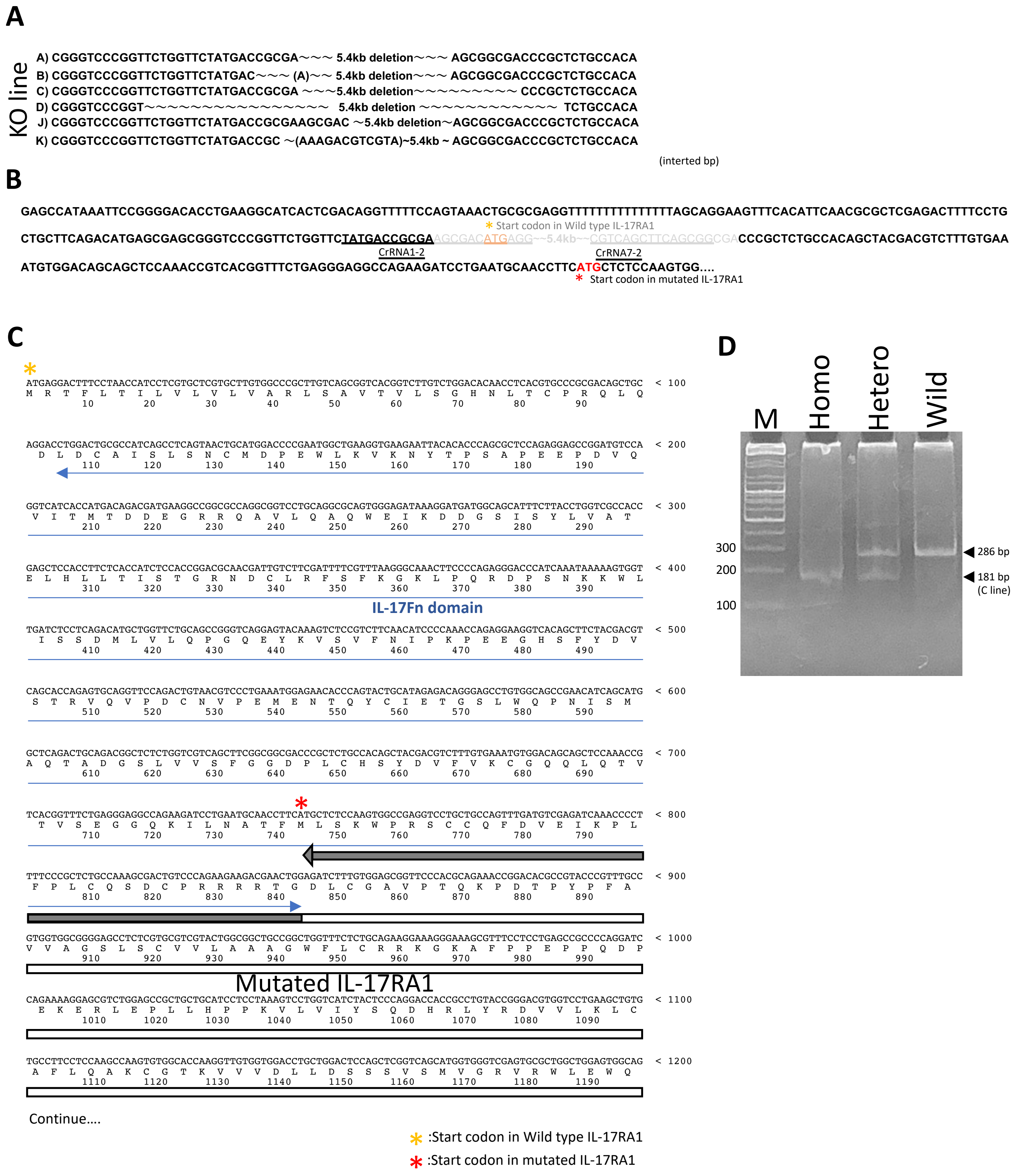

Fig. S2. The mutation start and end point of mutated *il17ra1*. (A) Overall, we obtained six variants of the mutant. Mutant line B and K were inserted the nucleotide (KO line B: 1 bp inserted, KO line K: 12 bp inserted.). All mutations in KO line started the up-stream of start codon of WT *il17ra1* (B) and predicted coding sequence of mutated *il17ra1* in each KO line are described (C). (D) Two forward primers and a reverse primer used for confirmation of genotype [F1-R1; 286 bp (amplification of the partial region of WT *il17ra1*), F2-R1; 181 bp (amplification of the partial region of mutated *il17ra1* (line C)].

Fig. S3.

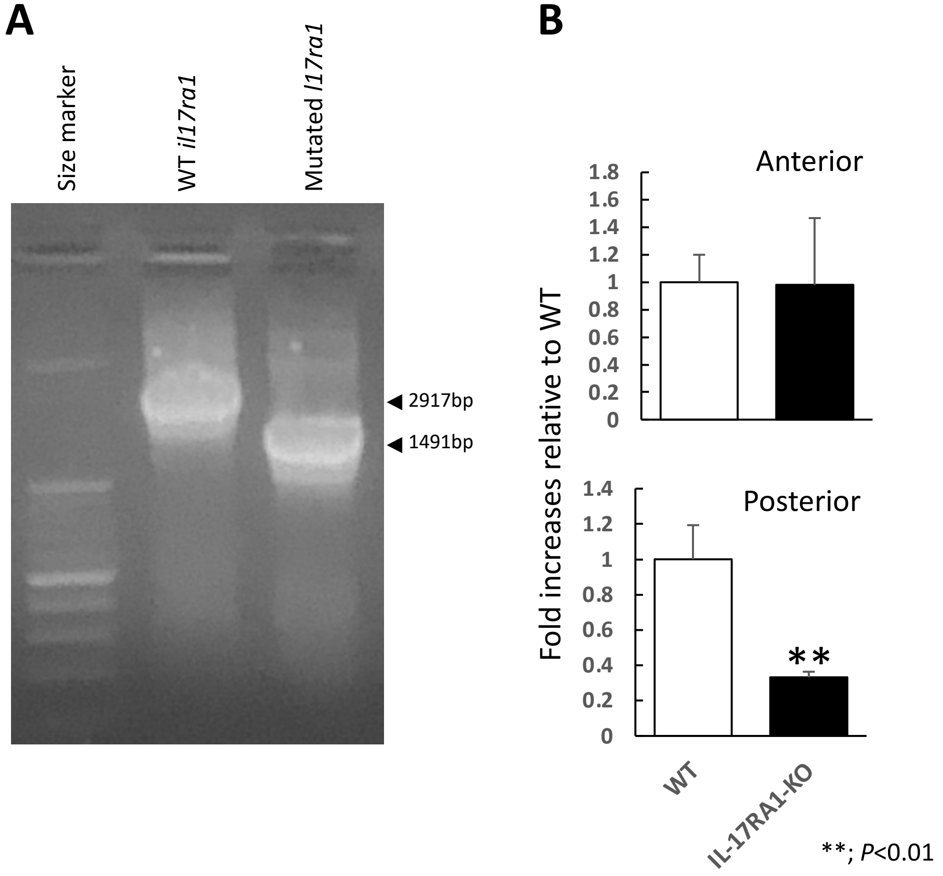

Fig. S3. The ORF comparison of WT and mutated *il17ra1* and its gene expression level in each section of the intestine. (A) The PCR product size of each ORF in WT and mutated *il17ra1*. The mutated ORF sequence was confirmed by read sequencing and WT *il17ra1* was not amplificated in the cDNA sample of IL-17RA1-KO. (B) The comparison of the gene expression level of *il17ra1* in anterior and posterior intestine of WT and IL-17RA1-KO medaka. $^{**}P < 0.01$, $^{*}P < 0.05$ (Student's *t*-test). Data are from one experiment with three individual fish (n = 3).

Fig. S4.

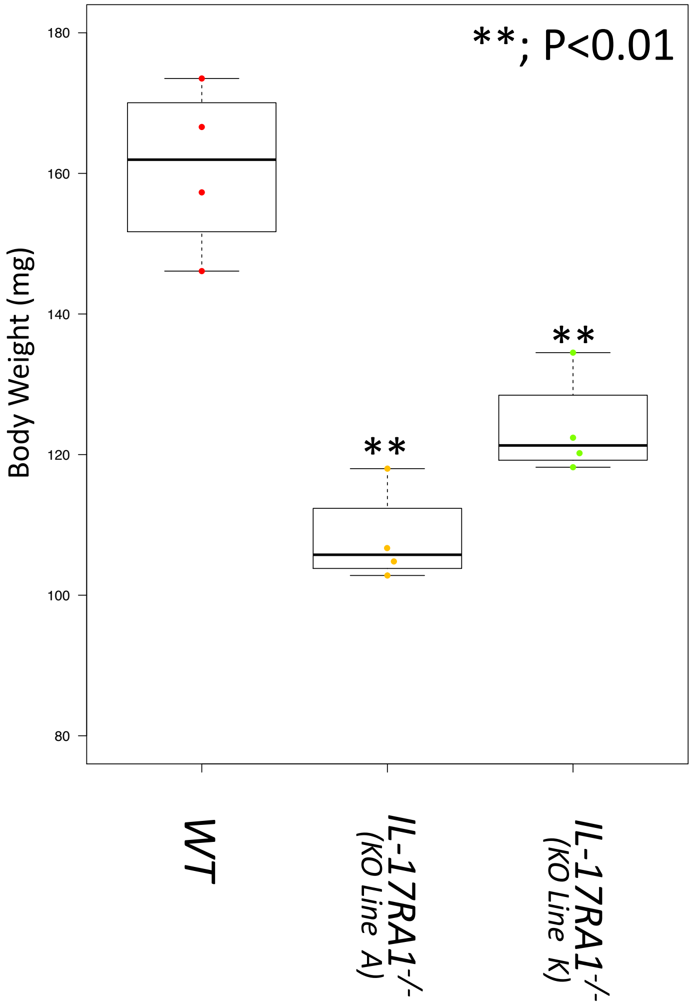

Fig. S4. The weight losses seen in other lines (KO lines A and K) homozygous mutant of IL-17RA1-KO medaka. In 4 months after hatching, body weight was measured (n=4).

Fig. S5.

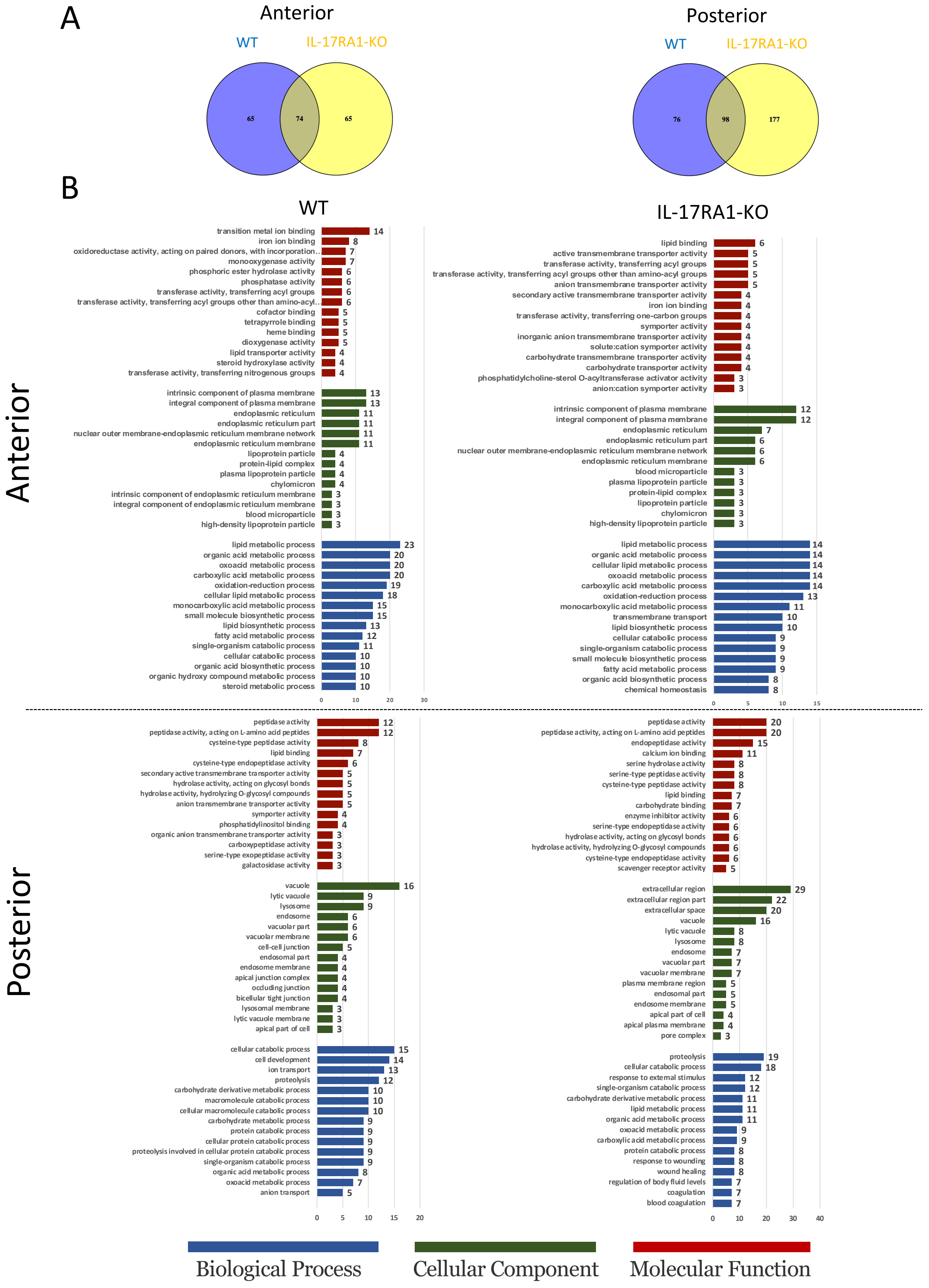

Fig. S5. The enrichment of gene ontology (GO) classification of the differently expressed genes (DEGs) comparing between anterior and posterior intestines. Significant different genes (*P*<0.05) were extracted as DGEs. (A) In WT's comparison of anterior and posterior intestines, and IL-17RA1-KO's comparison of anterior and posterior intestines, anterior 74 genes and posterior 98 genes were consensus DEGs in both of WT and KO. (B) Anterior intestine develops the expression of lipid metabolism-related genes.

Fig. S6.

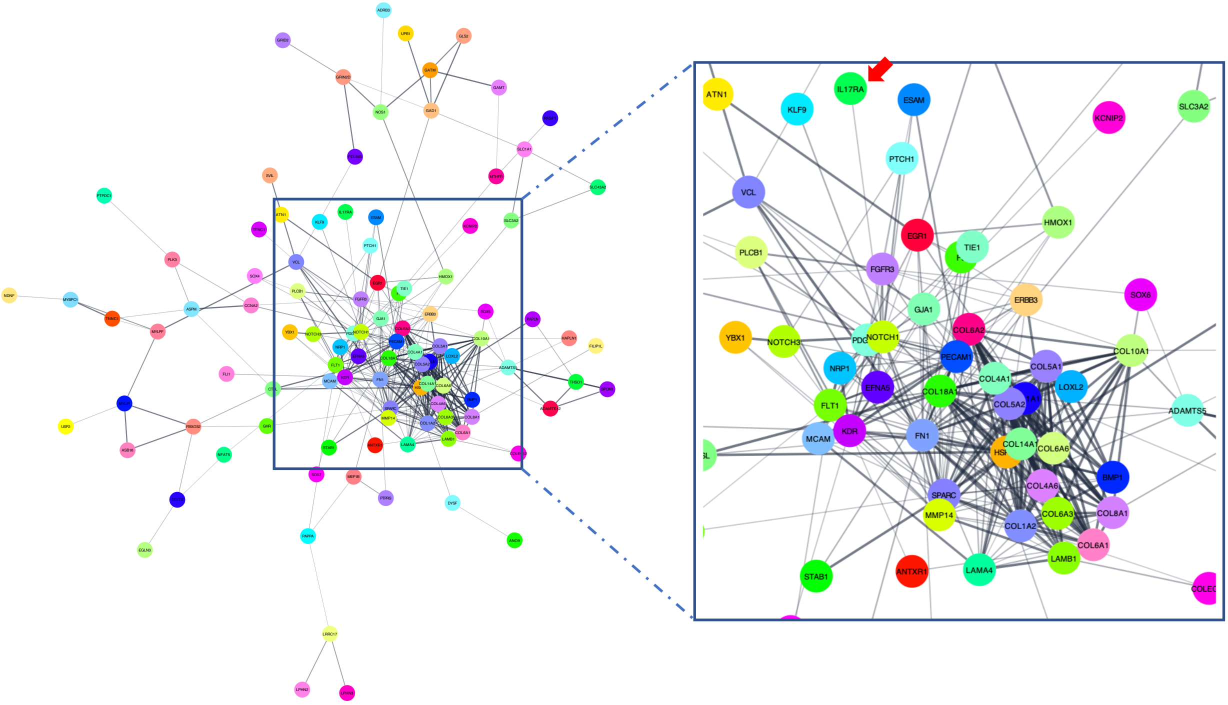

Fig. S6. The prediction of the interaction among the DEGs up-regulated in IL-17RA1-KO with IL-17RA. Interaction network of these genes as defined by Cytoscape's software STRING. Of 167 DEGs, 102 genes formed the most complex cluster and contained IL-17RA. Red arrow shows IL-17RA.

Fig. S7.

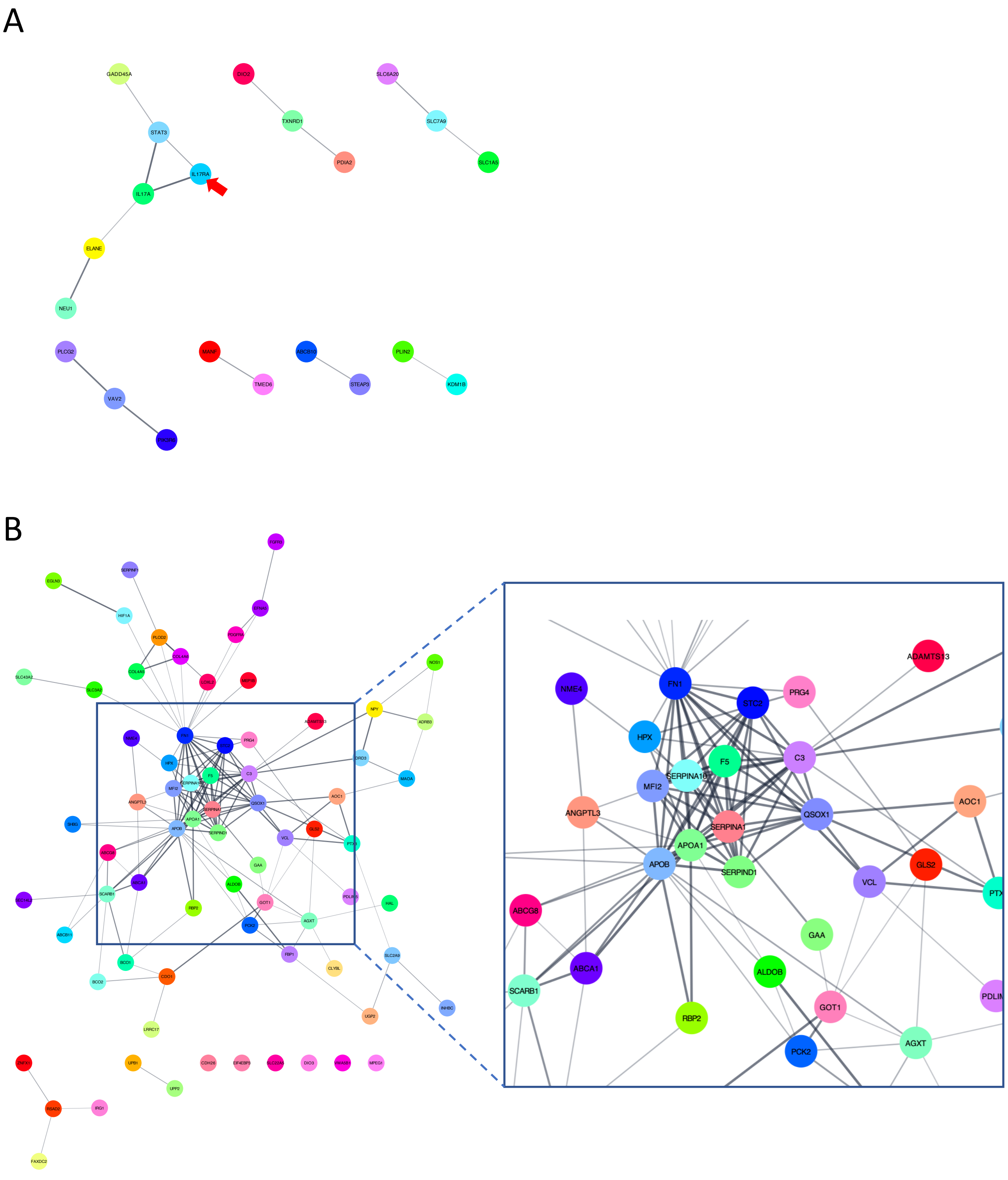

Fig. S7. The prediction of the interaction between posterior DEGs in IL-17RA1-KO and IL-17RA. Interaction network of these genes as defined by software STRING in Cytoscape. (A) Down-regulated DEGs in the posterior intestine of IL-17RA1-KO. IL-17RA containing cluster contains IL-17 signaling related genes such as the IL-17A/F1 ligands and transcriptional factor STAT3. Red arrow shows IL-17RA. (B) Up-regulated DEGs in the posterior intestine of IL-17RA1-KO. The up-regulated DEGs in the posterior intestine of IL-17RA1-KO did not form a cluster with IL-17RA.

Fig. S8.

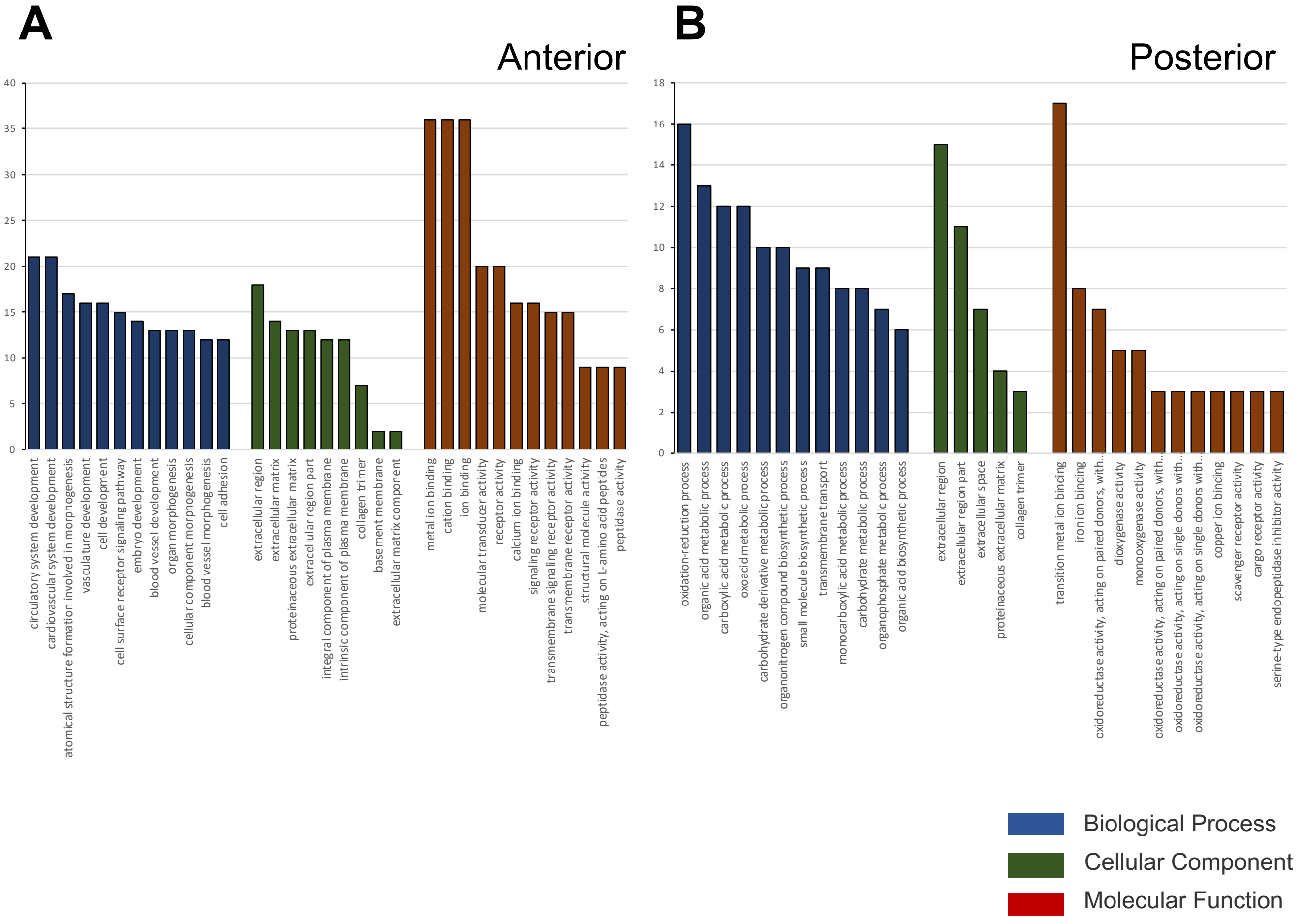

Fig. S8. Top 12 classification of GO enrichment of up-regulated DEGs in the anterior (A) and posterior intestines (B) of IL-17RA1-KO.

Fig. S9.

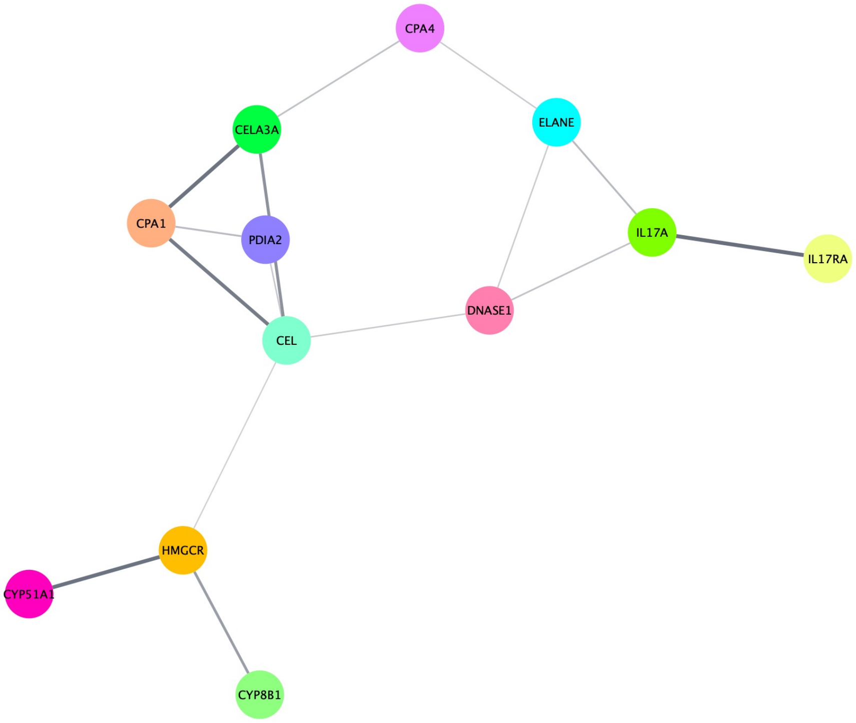

Fig. S9. The prediction of the interaction among DEGs down-regulated commonly in IL-17A/F1-KO and IL-17RA1-KO with IL-17A and IL-17RA. Interaction network of these genes as defined by Cytoscape's software STRING. Of 17 DEGs, 12 genes formed the most complex cluster containing IL-17A and IL-17RA.
